# Stabilisation of the hexasome intermediate during histone exchange by yeast SWR1 complex

**DOI:** 10.1016/j.molcel.2024.08.015

**Published:** 2024-09-02

**Authors:** Adam S.B. Jalal, Paul Girvan, Eugene Y.D. Chua, Lexin Liu, Shijie Wang, Elizabeth A. McCormack, Michael T. Skehan, Carol L. Knight, David S. Rueda, Dale B. Wigley

**Affiliations:** 1 Section of Structural Biology, Dept. Infectious Disease, Faculty of Medicine, Imperial College London, London SW7 2AZ, U.K; 2 Single Molecule Imaging Group, MRC Laboratory of Medical Sciences, Du Cane Road, London W12 0HS, U.K; 3 Section of Virology, Dept. Infectious Disease, Faculty of Medicine, Imperial College London, Du Cane Road, London W12 0HS, U.K

## Abstract

The yeast SWR1 complex catalyses the exchange of histone H2A/H2B dimers in nucleosomes, with Htz1/H2B dimers. We use cryo-electron microscopy to determine the structure of an enzyme-bound hexasome intermediate in the reaction pathway of histone exchange in which a H2A/H2B dimer has been extracted from a nucleosome prior to insertion of a dimer comprising Htz1/H2B. The structure reveals a key role for the Swc5 subunit in stabilising unwrapping of the DNA from the histone core of the hexasome. By engineering a crosslink between an Htz1/H2B dimer and its chaperone protein Chz1, we show that this blocks histone exchange by SWR1 but allows the incoming chaperone-dimer complex to insert into the hexasome. We use this reagent to trap a SWR1/hexasome complex with an incoming Htz1/H2B dimer that shows how the reaction progresses to the next step. Taken together the structures reveal insights into the mechanism of histone exchange by SWR1 complex.

## Introduction

**Figure 1:**
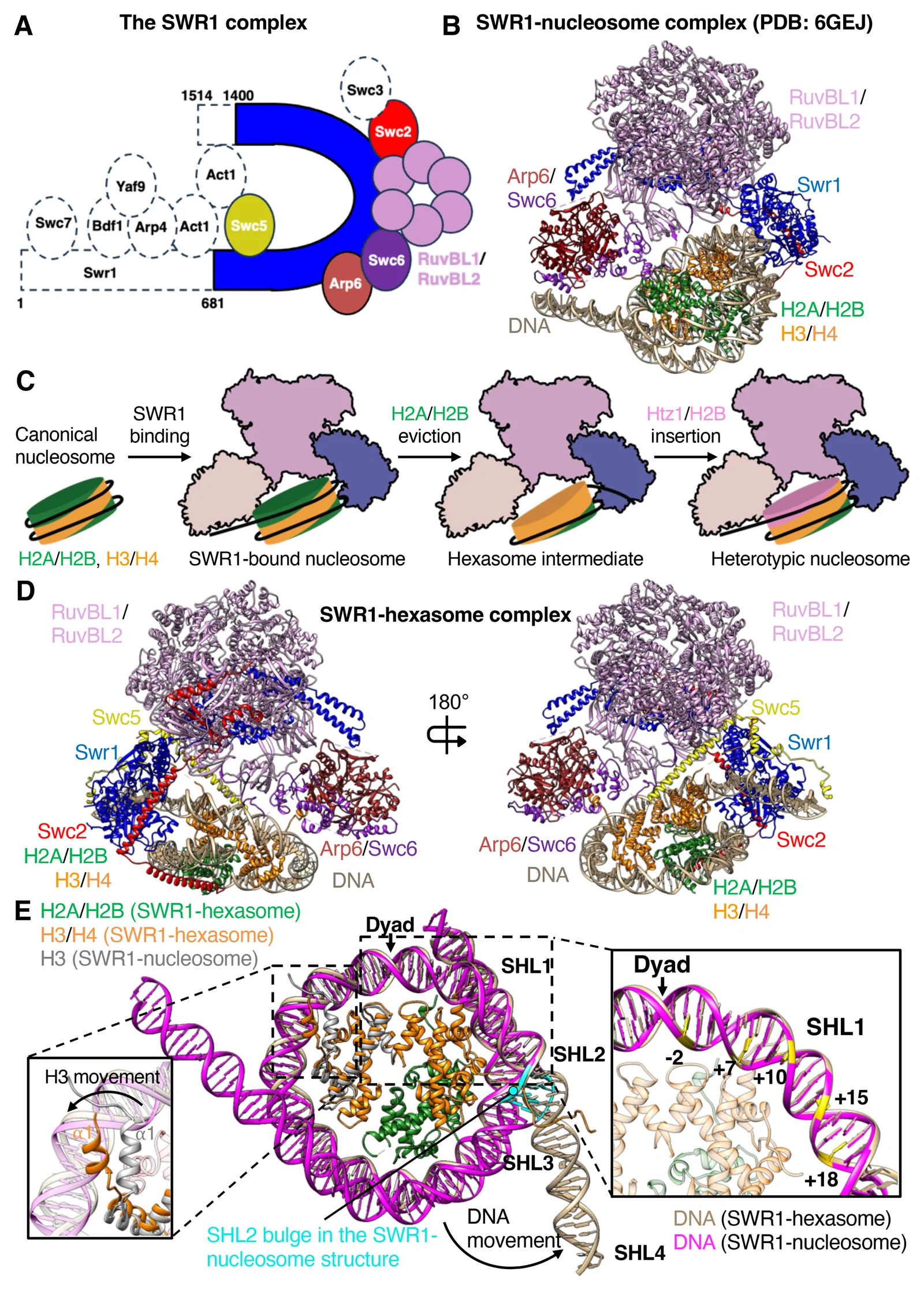
Overview of the SWR1-hexasome complex architecture and distortions introduced into the hexasome by SWR1. **(A)** Cartoon representation of the yeast SWR1 complex. Coloured subunits represent those that are ordered in the SWR1-hexasome structure, while subunits depicted with a dotted outline represent the subunits that were not visible. **(B)** Overview of the SWR1-nucleosome complex (PDB:6GEJ). **(C)** Cartoon showing the multiple steps involved in SWR1-mediated histone exchange to generate a heterotypic nucleosome consisting of one dimer each of H2A/H2B and Htz1/H2B. A second similar step (not shown in this cartoon) then occurs to generate the homotypic nucleosome with both dimers exchanged. **(D)** Overview of the SWR1-hexasome complex. **(E)** DNA at super helical location (SHL) 2 is unwrapped in the SWR1-bound hexasome structure. Structural comparison between hexasome and the SWR1-bound nucleosome (PDB: 6GEJ). The SWR1-nucleosome structure was superimposed onto the SWR1-hexasome structure using Swr1 HD1-HD2 domains as reference. For clarity, only the nucleosomal DNA and one H3 histone is shown in the SWR1-bound nucleosome structure as a comparison with the hexasome H3 in the same position. The close up view on the left shows the α1 helix of histone H3 shifting position in the SWR1-bound hexasome. The close up on the right shows that the DNA remains unchanged between the dyad and SHL2 of the wrap in the SWR1-bound hexasome.

SWR1 complex in yeast catalyses the exchange of H2A/H2B dimers for those comprising Htz1/H2B^1,2,3^. In humans, this task is undertaken by the SRCAP^4^ and TIP60^5^ complexes. Nucleosomes modified in this way are involved in regulation of processes such as transcription and DNA repair^6^. SWR1 complex is a member of the INO80 family of remodellers^7^. The complex comprises fourteen subunits and the cryo-electron microscopy (cryo-EM) structure of SWR1 bound to a nucleosome has been reported previously^8^ (Figure 1A & 1B). The main body of the complex interacts with the bound nucleosome. The subunits in this part of the structure are assembled around a core of heterohexameric AAA+ proteins, RuvBL1 and RuvBL2. The Arp6/Swc6 module, Swc2, Swc3 and Swr1 subunits interact with this core which together are termed Subcomplex 2 (SC2). Several subunits form a part of the complex termed Subcomplex 1 (SC1 – the N-terminal region of Swr1 with subunits Bdf1, Yaf9, Swc4, Swc7, Arp4 and two actin molecules) that is disordered in the structure. Additionally, the Swc5 subunit was not visible in the structure. Biochemical data^9^ suggest that Swc5 might bridge the SC1 and SC2 components.

The reaction pathway for SWR1-mediated histone exchange is shown in Figure 1C. The replacement of the two histone dimers in a nucleosome is stepwise and sequential^10,11^. For each histone exchange event to take place, a dimer has to be removed to create a hexasome intermediate. How this intermediate is produced appears to involve ATP-dependent DNA translocation, or distortion, to unwrap the DNA from the histone core to facilitate release of a specific dimer but without overall sliding of the nucleosome^2,10,11^.Work on the steps leading up to dimer extraction have started to reveal how this process takes place, but if (or how) the hexasome intermediate might be stabilised by SWR1 is not known.

In order to address this question, we first determined a structure of a complex between SWR1 and a hexasome. The structure reveals a role for the Swc5 subunit in stabilising both the unwrapped DNA and the exposed histone core. In a second structure, we trap the complex with an incoming chaperone-bound dimer and we show how SWR1 also stabilises the hexasome with the Htz1/H2B dimer bound in position but before the DNA is rewrapped around the histone core. These structures show how SWR1 protects and stabilises the hexasome intermediate and then facilitates binding of the incoming dimer.

## Results

### SWR1-hexasome structure

The structure of SWR1 complex bound to a yeast hexasome, comprising a H3/H4 tetramer and a single H2A/H2B dimer (referred to hereafter as hexasome), was determined using cryo-electron microscopy at a resolution of 3.5Å in the presence of the non-hydrolysable ATP analogue ADP.BeF3 without the need of crosslinking (Figures 1D, S1, S2, Table S1 & Movie M1). The main features of the SWR1 complex were very similar to those in the complex with a nucleosome^8^ (Figure 1B). The SC1 component, comprising the N-terminal region of Swr1 and a number of other subunits, is disordered as observed in other structures^8^.

However, there are significant differences between the hexasome and nucleosome components bound to SWR1 complex. Most obvious is the DNA wrap around the histone core. In the SWR1-nucleosome complex^8^, approximately 2.5 turns of DNA are unwrapped compared to a canonical nucleosome^16^. This unwrapped DNA is stabilised by interaction with the Arp6/Swc6 module. Additionally, a small bulge is introduced due to a portion of the DNA, from the motor domains to the entry point, being shifted by one base pair as a result of binding of nucleotide (ADP.BeF3) to the motor domains. This suggests a translocation step size of one base per ATP as observed for a number of other helicase/translocases^13^. Previous studies have shown that binding and or hydrolysis of ATP by the complex cause dynamic fluctuations in DNA consistent with partial unwrapping^14–16^.

**Figure 2:**
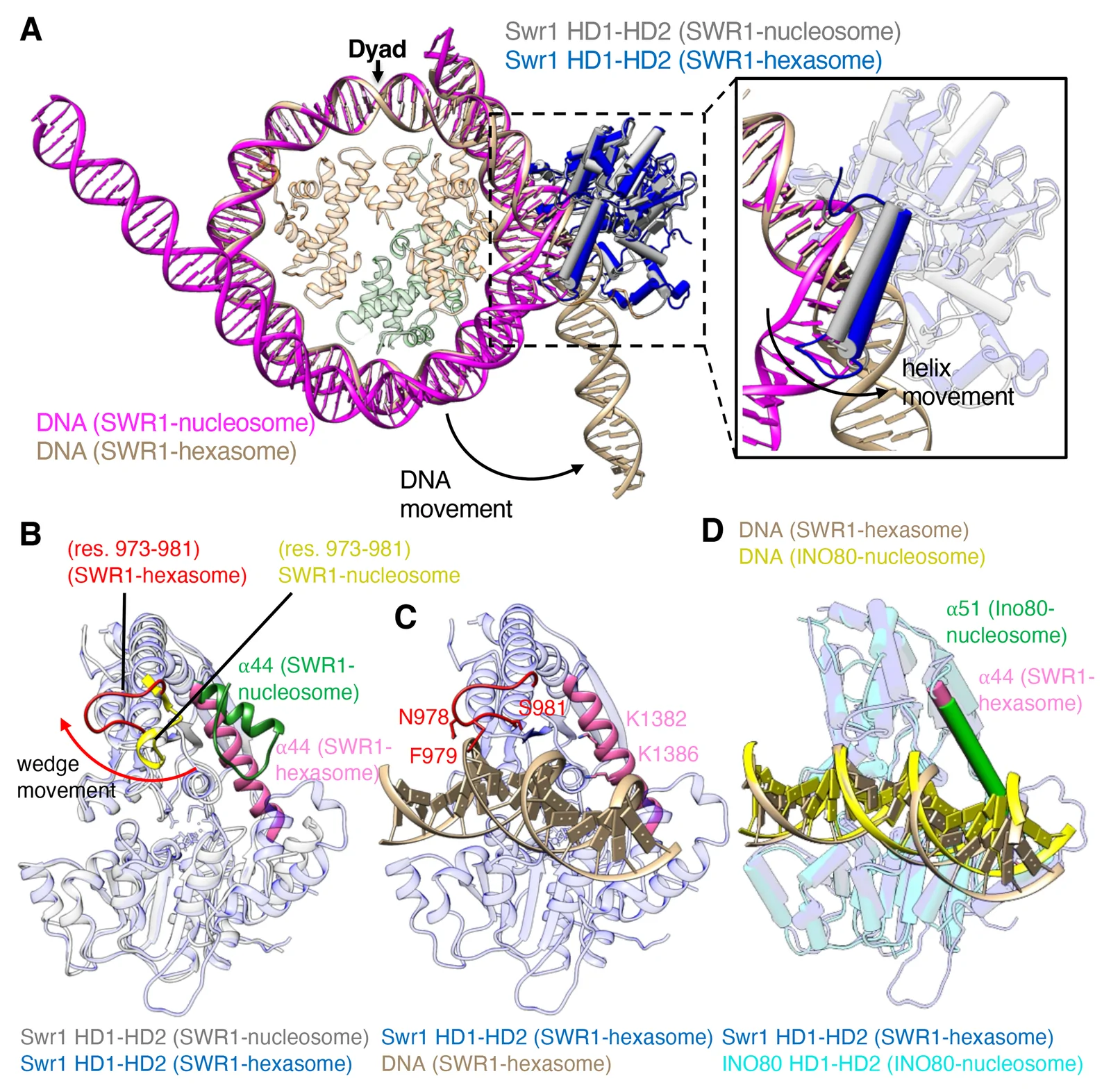
Structural changes of the Swr1 motor domains in the hexasome complex. **(A)** The SWR1-nucleosome structure (PDB:6GEJ) and SWR1-hexasome structure were superimposed upon the hexasome core particle. For clarity, in the SWR1-hexasome structure only the histones, hexasomal DNA and Swr1 HD1-HD2 are shown, while in the SWR1-nucleosome structure, only the nucleosomal DNA and Swr1 HD1-HD2 are shown. The inset is a close up view showing the helix over SHL2 moving in the same direction as the hexasomal DNA at SHL2. **(B)** Swr1 conformational change in the nucleosome and hexasome bound states. **(C)** Swr1 conformational changes engage the unwrapped hexasomal DNA at SHL2. The wedge (residues 973-981) sits against the minor groove of the unwrapped SHL2 DNA in the hexasome-bound structure. While the conformational change in α44 creates a new DNA-binding surface that stabilises the unwrapped hexasomal DNA. **(D)** Swr1 motor domains engage DNA in an analogous manner to those of Ino80 in the nucleosome bound state. The Ino80 subunit HD1-HD2 domains (INO80-nucleosome) were superimposed onto the Swr1 HD1-HD2 domains (SWR1-hexasome) (PDB:6HTS).

In the transition from nucleosome to hexasome, this unwrapping has been taken much further so that more than five turns of duplex DNA are now peeled away from the hexasome surface (Figure 1B & 1D, Movies M1 & M2). This unwrapping of the DNA exposes one of the dimers in a nucleosome and this dimer is the one that is lost in forming the hexasome (Figure 1C & Movie M2). Additionally, α1 of the surface exposed histone H3 shifts towards the SWR1 complex (Figure 1E & Movie M2). Structural comparison between SWR1-bound hexasome and a free hexasome^17^ demonstrates that these conformational changes are induced by SWR1 (Figure S3). Although our structure does not provide details of how SWR1 progresses from the nucleosome to hexasome-bound states, this has been shown to be the result of multiple cycles of ATP hydrolysis and DNA translocation or distortion by the motor domains^11,15^. However, the structure does show that once the dimer has been exposed and displaced from the histone core, the DNA wrap is held open by the SWR1 complex until the extracted dimer has been replaced with an Htz1/H2B dimer. The large segment of unwrapped DNA is constrained via the action of two principal subunits – the Swr1 motor domains and Swc5. The Swr1 motor domains are located at exactly the same number of base pairs from the dyad on the DNA as seen in the nucleosome complex structure but now have shifted away from the histone core to hold the wrap away from this surface (Figures 1E & 2A). Additionally, a loop (residues 973-981) that sits in between the two helices responsible for the DNA bulge in the SWR1-nucleosome structure (Figure 1E), now tucks into the minor groove of the unwrapped DNA at SHL2 (Figures 2B & S3). This acts as a wedge which prevents DNA from rewrapping in the hexasome, priming the hexasome in a conformation compatible with dimer deposition (Figures 2B & S3). Furthermore, the hexasome intermediate is stabilised by conformational changes in the Swr1 motor, generating a new DNA-binding site that engages the unwrapped DNA in a manner that is more similar to that observed for the Ino80 subunit bound to either nucleosome^18,19^ or hexasome^20,21^ substrates (Figures 2B-2D & S3), than it is to the SWR1-nucleosome complex^8^.

### The Swc5 subunit plays a key role in stabilisation of the hexasome intermediate

**Figure 3:**
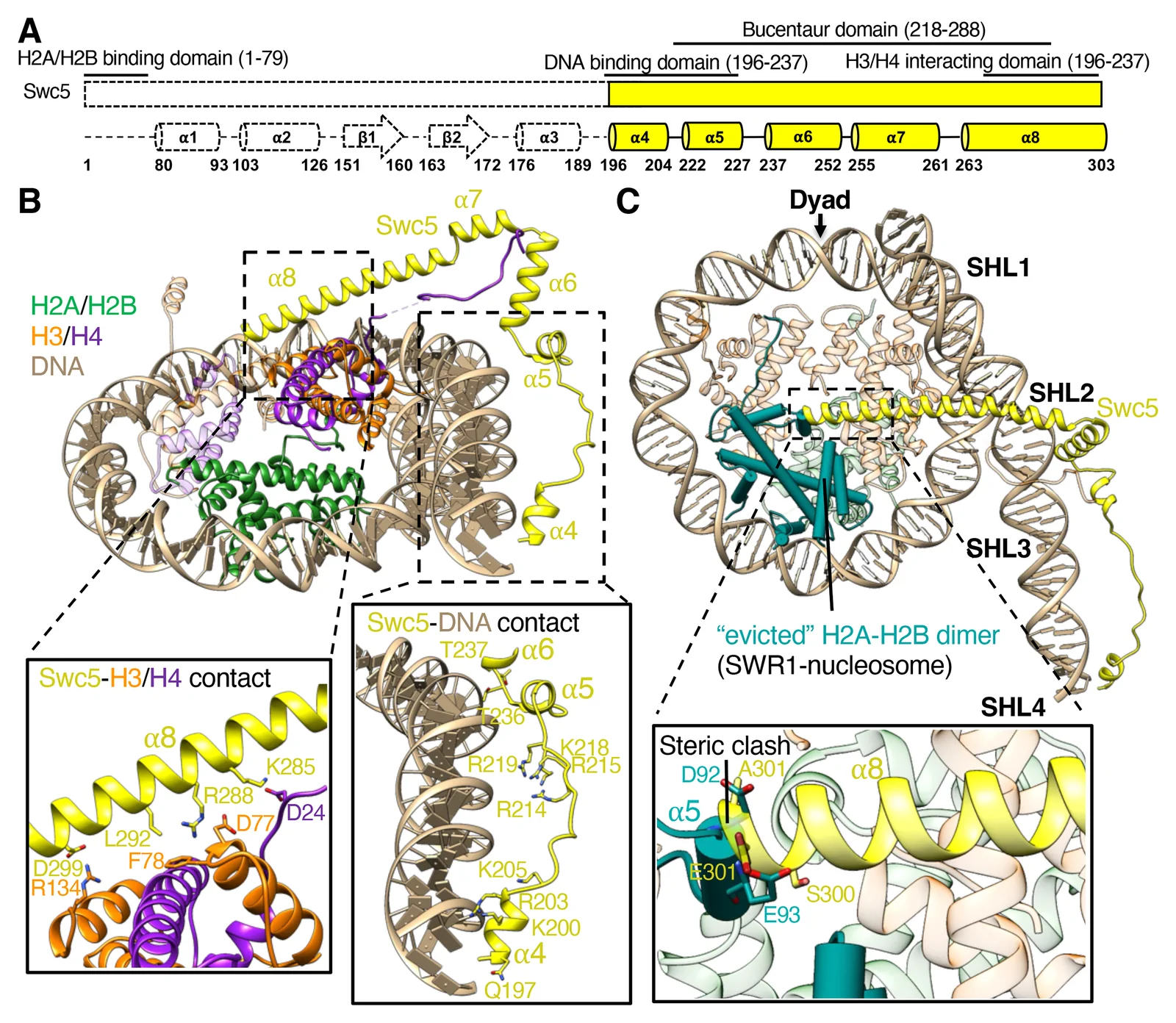
Detailed interactions between the Swc5 subunit and the hexasome. **(A)** Linearized cartoon of the Swc5 subunit. The built (solid) and AlphaFold predicted (dashed) secondary structure is represented as a cartoon below. **(B)** Swc5 forms an extended structure that interacts with multiple elements of the hexasome. Close up views detail the interactions between Swc5 and (left) H3/H4 histone (only Swc5 and the interacting H3/H4 histones are shown), and (right) unwrapped hexasomal DNA. **(C)** Swc5 clashes with the position that would be occupied by the “evicted” H2A/H2B dimer. To model the position of the “evicted” dimer H2A-H2B, the SWR1-nucleosome structure was superimposed onto the SWR1-hexasome structure using Swr1 HD1-HD2 domains as reference (PDB:6GEJ). Inset shows a close up of this steric clash. The last four residues of Swc5 clash with the acidic patch on the “evicted” H2A/H2B dimer.

The Swc5 subunit is essential for enzyme activity^9,22,23^ but was not ordered in the structure of the SWR1-nucleosome complex^8^. By contrast, we now observe additional density right at the heart of the complex with the hexasome. Using Alphafold^24^ to predict the structure, we were able to assign this density as Swc5 (Figures 1D, 3A, 3B, S4, Movie M1). The predicted fold of the C-terminal region of Swc5 fitted into the density with just minor adjustments (Figures 3B & S4). Once this region (residues 237-303) was fitted, we were able to extend the structure further (residues 196-236) across a region of the Swc5 structure that was poorly predicted. The Swc5 subunit wraps across the surface of the Swr1 motor domains (Figure 1D & Movie M1) before making extensive contacts with the unwrapped portion of the DNA, covering a region extending across around two turns of the duplex from SHL2 to SHL4 (Figures 3B, 3C & S4). Specifically, several Swc5 residues (located between 197-237; Figure 3 & Movie M1) interact with the unwrapped DNA, and mutations in these residues have been shown to impair SWR1 activity^23^. Previous work has identified a role for Swc5 in stimulating ATPase activity of Swr1^23^ and the structure shows this is now well positioned for this activity. Together with the motor domains of the Swr1 subunit, the Swc5 DNA contacts help to stabilise the unwrapped DNA and hence the hexasome intermediate.

Additionally, the Swc5 subunit makes extensive contacts with the exposed histone protein interface (Figures 3B & 3C). This contact region involves residues in the C-terminal helix (α8) of Swc5 that make direct contacts with histones H3 and H4. These contacts help to pull the empty dimer site in the hexasome up against the protein surface, to protect and stabilise it, until a new dimer is ready to be positioned. While the position of Swc5 in SWR1-bound nucleosome is uncertain, the H2A/H2B dimer must first be evicted before Swc5 can bind the exposed histone surface (Figure 3C). Biochemical evidence demonstrated that Swc5 alone does not bind to the nucleosome^23^, suggesting that Swc5 recognises and stabilises the structural changes in the SWR1-bound hexasome.

In order to confirm the role played by Swc5, we prepared a SWR1 complex lacking this subunit (ΔSwc5). Swc5 is one of the few subunits that can be deleted from the complex without the loss of other subunits^9,22,23^. We then determined the cryo-EM structure of the ΔSwc5 complex with a hexasome in the presence of the non-hydrolysable ATP analogue ADP.BeF3 without the need of crosslinking (Figures S1, S2, S4 & Table S1). The mutant complex binds hexasomes a little less tightly so we had fewer complexes on grids than for the full complex, which might be expected since Swc5 makes significant contacts with the hexasome (see above). Similarly, as expected, the most obvious difference in the ΔSwc5 complex structure was the lack of density that we had assigned as Swc5 in the full complex (Figure S4), notably the long helix that interacts across the hexasome surface. In addition, we observed significant changes in the degree of unwrapping of DNA from the hexasome compared to the full complex (Figure S4). Deletion of the Swc5 subunit reduces the unwrapping to an intermediate stage between a nucleosome bound to Swr1 and that seen in a hexasome. Interestingly, the degree of unwrapping we observe in the ΔSwc5 SWR1 bound hexasome is very similar to that observed for a hexasome bound to INO80^20,21^ (Figure S4). Consequently, the structure revealed additional information supporting the role we propose for Swc5 in stabilising the large unwrapping of DNA from the hexamer histone core to facilitate exchange.

### Crosslinking of chaperone-dimer or nucleosome substrates block histone exchange by SWR1

**Figure 4:**
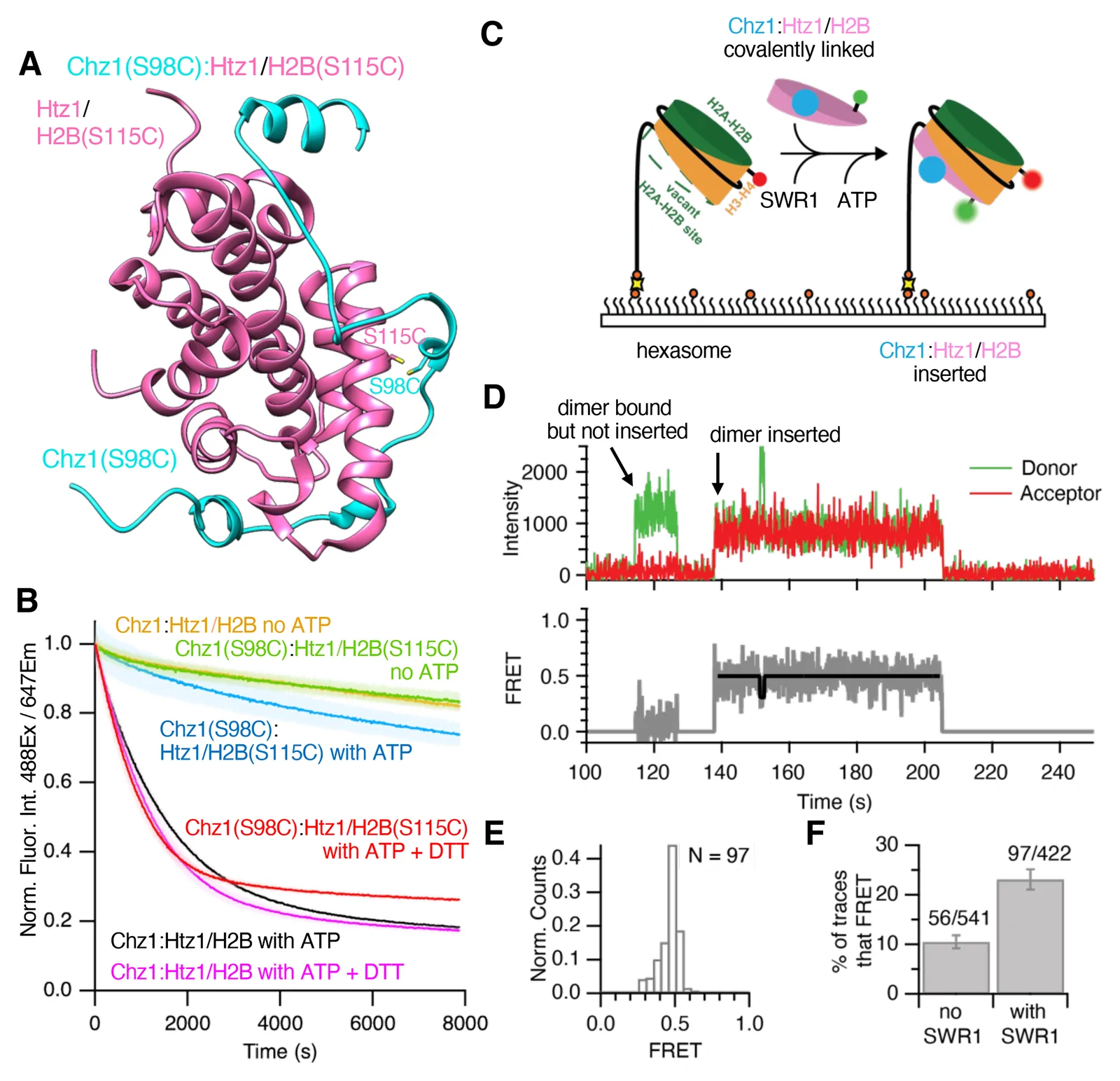
A covalently linked Chz1:Htz1-H2B complex traps SWR1-hexasome at an intermediate step of histone exchange. **(A)** The NMR structure of the Chz1:Htz1-H2B complex (PDB:2JSS). The point mutations introduced to generate the covalently linked Chz1(S98C):Htz1/H2B(S115C) complex are overlaid. **(B)** Bulk FRET-based histone exchange assay following loss of FRET. The covalent bond linking Chz1(S98C):Htz1/H2B(S115C) prevents SWR1 from displacing Chz1, thus inhibiting SWR1-mediated histone exchange. Reducing the covalent bond between Chz1(S98C) and Htz1/H2B(S115C), by pre-incubating with DTT, restores SWR1-mediated histone exchange to wildtype levels. H2A/H2B dimers within nucleosomes were labelled as described in Star Methods. **(C)** Cartoon of the single-molecule assay. Hexasomes, with a FRET acceptor on the short end of the DNA linker, are immobilized on a microscope slide. SWR1, ATP and covalently linked Chz1(S98C)-Htz1/H2B(S115C) dimers are flowed in. Labelling is as shown in the figure and described in Star Methods. **(D)** Example trajectory from a single hexasome showing donor and acceptor intensity (top) and FRET (bottom). Individual events can be distinguished including dimer binding (without insertion, t = 120 s) and insertion into the hexasome (t = 140 s). The observation of binding but not insertion suggests either a second dimer binding site or extraneous binding to (for example) the DNA linker region (t = 120 & 150 s). The drop in FRET at 205 s indicates either photobleaching or dimer dissociation. **(E)** FRET histogram showing that the inserted dimer is positioned in the vacant distal H2A/H2B dimer site. **(F)** Insertion of an Htz1/H2B dimer into a hexasome is more efficient in the presence of SWR1.

Having determined the structure of the hexasome intermediate, we wanted to obtain information about the next step of the reaction which involves insertion of a new Htz1/H2B dimer into the space created by the loss of the outgoing H2A/H2B dimer. However, if dimer were added directly to the SWR1-hexasome complex then we had no way to prevent the reaction progressing to completion. In order to trap this next intermediate some intervention was required. *In vivo*, extranucleosomal Htz1/H2B dimers are complexed with either the general histone dimer chaperone Nap1^2,3^ or the specific Htz1/H2B chaperone Chz1^25^, to reduce non-specific binding of histones to DNA. Structures of a complex between Chz1 and an Htz1/H2B dimer have been determined previously^26,27^. Using these coordinates as a guide, we identified residues to mutate to cysteine to engineer an internal disulphide crosslink that covalently bonds the Chz1 protein to the Htz1/H2B dimer (Figures 4A, S5 & S6). When used in place of Chz1-dimer complexes, the histone exchange reaction was blocked unless a reducing agent (DTT or TCEP) was added to break the crosslinks (Figures 4B, S5 & S6). In order to investigate further the consequences of forming this crosslink between the chaperone and the dimer we utilised single-molecule methods (Figure 4C). By labelling hexasomes, we were able to monitor association of dimer-chaperone complexes by FRET (Figures 4D & 4E). Furthermore, we were able to observe a higher proportion of hexasome-dimer-chaperone complexes in the presence of SWR1 complex (Figure 4F) showing not only that the crosslinked histone dimer-chaperone binds to SWR1 but also that this complex is stabilised on-enzyme by SWR1 so is a *bona fide* proxy for the hexasome:dimer intermediate in the reaction cycle.

We also created another reagent in which an internal disulphide crosslink was introduced between histone H2A/H2B dimers to prevent dimer removal from the nucleosome (Figure S5). As expected, this nucleosome was unable to undergo SWR1-mediated histone exchange in the absence of a reducing agent but showed wildtype activity when DTT was added (Figure S5). As a control for integrity of the crosslinked nucleosome, we used INO80 complex to see if it could bind and slide the nucleosome. Human INO80 complex was able to slide the nucleosome equally well both in the presence and absence of DTT confirming that the crosslink does not disturb the structure (Figure S5), as shown previously for the yeast enzyme^21^.

### Structure of SWR1-hexasome-Chz1-dimer complex

**Figure 5:**
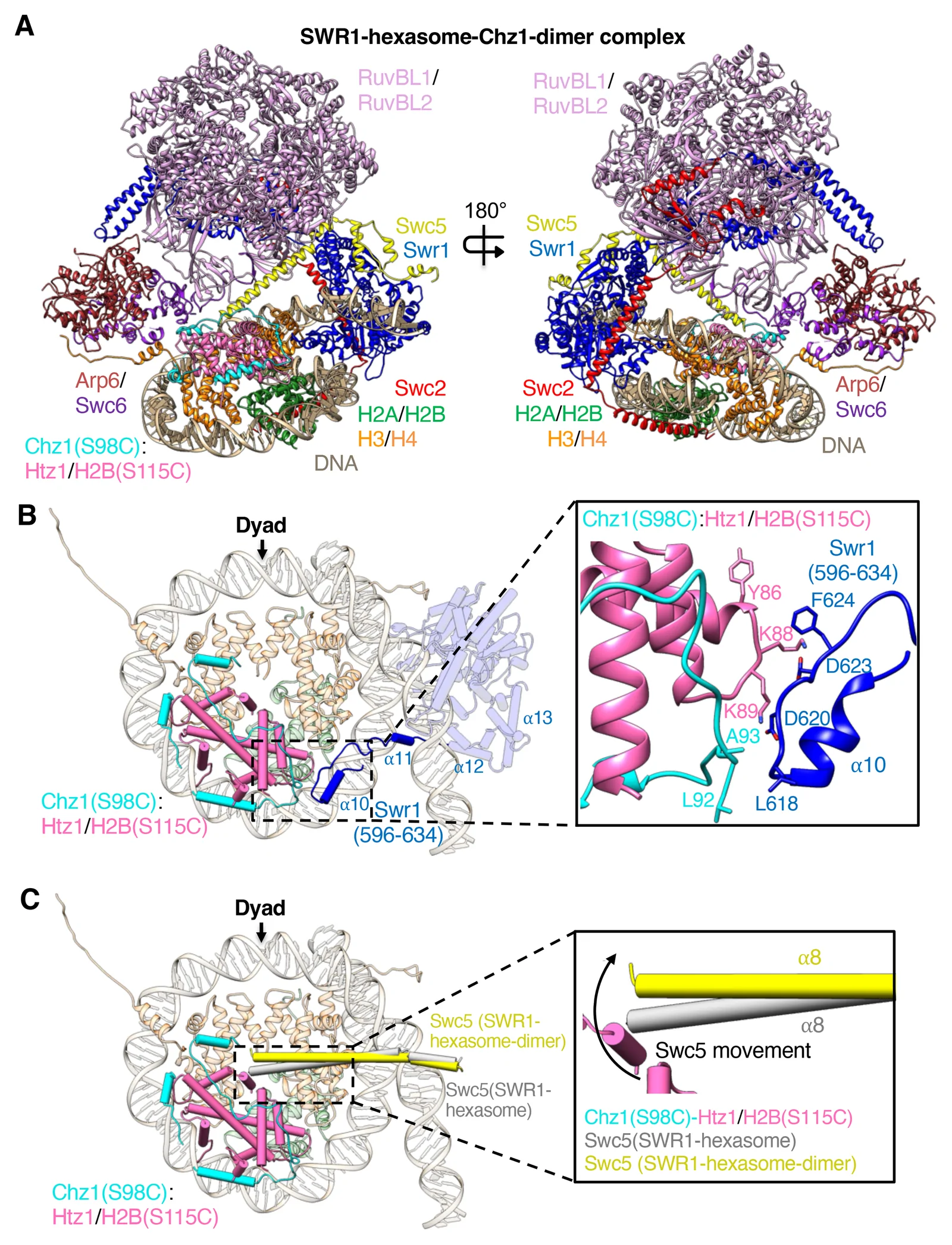
Structural changes in the SWR1 stabilise Chz1:Htz1-H2B insertion into the hexasome. **(A)** Overview of the SWR1-hexasome-Chz1-dimer complex. **(B)** The Swr1 subunit recognizes the incoming dimer in the SWR1-hexasome-Chz1-dimer structure. Inset shows details of this interaction but, for clarity, with only the Chz1(S98C):Htz1/H2B(S115C) and Swr1 (residues 596-634) shown. **(C)** Helix α8 of Swc5 shifts in position to accommodate the incoming the Chz1(S98C):Htz1/H2B(S115C) complex. Inset shows the movement of α8 of Swc5. The SWR1-hexasome structure was superimposed onto the SWR1-hexasome-Chz1-dimer structure using the hexasome core particle as reference. For clarity, in the SWR1-hexasome-Chz1-dimer structure only the histones, Chz1(S98C):Htz1/H2B(S115C), hexasomal DNA and Swc5 (α7-α8) are shown, while in the SWR1-hexasome structure, only Swc5 helices α7-α8 are shown for comparison.

Having established that SWR1 complex can recognise and incorporate a crosslinked Chz1-dimer into a hexasome, we determined the structure of this blocked intermediate bound to the SWR1-hexasome complex at 3.8Å resolution in the presence of the non-hydrolysable ATP analogue ADP.BeF3 without the need of crosslinking (Figures 5A, S1, S2, S6, Table S1 & Movie M3). Overall, the architecture of the complex is very similar to that of the SWR1-hexasome complex (Figure S6). In particular, the unwrapping and stabilisation of the DNA are similar in both structures.

Additionally, there is clear density for the bound covalently linked Chz1:Htz1/H2B complex, located at the position expected for an incoming histone dimer (Figures 5B & S6, Movie M3). The resolution of this component is lower than other parts of the structure so detailed model building beyond the residues observed in the reported structures^26,27^ was initially difficult, nevertheless, the use of AlphaFold multimer enabled us to assign density corresponding to additional residues of Chz1 that were omitted in the NMR and crystal structures (Figures 5B & S6, Movie M3). Chz1 binds across the DNA binding surface of the dimer^27^, preventing the DNA from rewrapping around the histone core, thereby trapping this next step in the reaction. Previous work has shown that Chz1 can be displaced from Htz1/H2B dimers by the Swc2 subunit^25^ although it has not been demonstrated whether this is required before or after dimer eviction. SWR1 can be pre-primed with Htz1/H2B dimers^22^ but assays conducted in the presence and absence of Chz1^22^ (Figure S6) show similar rates for histone exchange showing that the handover/removal of Chz1 is not rate limiting. The observation that the covalently linked Chz1:Htz1/H2B complex can bind to SWR1-hexasomes and that binding is stabilised by SWR1 complex (Figures 4D-4F) suggests it is possible for Chz1 to be displaced either to pre-prime SWR1 or after association with, or formation of, a hexasome. Either situation could require active displacement of Chz1 by Swc2^25^.

Another difference between the structures is in the interaction between the Swr1 motor domains and the DNA. In the chaperone-dimer bound complex, additional density can be observed extending from the N-terminus of the motor domains. Four short helical regions of Swr1 become ordered (α10-α13, residues 596-678), with α12 and α13 folding over the motor domains before the protein extends across the unwrapped DNA at the site where two turns of the DNA wrap would sit next to each other after re-wrapping (Figures 5B & S7). Consequently, binding of helices α12 and α13 of Swr1 would block re-wrapping of the DNA. The Swr1 helices upstream of the DNA contact (α10 & α11) extend across to contact the H2B component of the incoming dimer and the Chz1 subunit (Figure 5B). Thus, the presence of an incoming dimer is sensed by the Swr1 subunit directly. Mutation of the Swr1 residues involved in this contact region (D623A/F624A) have been shown to reduce binding of Htz1/H2B dimers by around 30-fold^28^. Furthermore, since there is also an interaction with the Chz1 and Swr1 in this structure, it may be that this is part of a mechanism to displace the Chz1 from the Htz1/H2B dimer along with a role played by the Swc2 subunit^25^. Consistent with this suggestion, a crystal structure of Swr1 (residues 599-627) complexed with Htz1/H2B^28^ shows that this region of Swr1 interacts with the Htz1/H2B dimer providing specificity for the interaction compared with H2A/H2B and overlaps with the Chz1 binding site^26^. However, this same region of Swr1 is interacting differently when Chz1 is prevented from displacement by crosslinking to the incoming dimer but is poised appropriately to contribute to the displacement (Figure 5B).

Previous studies on Swc5 have identified a motif (DEF/Y) in the N-terminal region of Swc5 that also interacts with histone dimers^23,29^ at a site that also overlaps with one of the interfaces between Chz1 and dimers^26,27^ and the interface with Swr1 described above^28^. This region is not ordered in our structure but is likely prevented from interacting with the incoming dimer by the presence of the crosslinked Chz1 since their binding sites overlap.

While the Swc5 subunit makes extensive contacts with the exposed histone protein interface in the SWR1-hexasome, these contacts are altered when Chz1:Htz1/H2B is deposited (Figure 5C). To accommodate these changes, helix α8 of Swc5 shifts position slightly, providing access for the incoming dimer. Deposition of free Htz1/H2B might then signal the release of the unwrapped DNA at SHL2 to initiate DNA rewrapping to form the heterotypic nucleosome.

### Comparison between SWR1-nucleosome, SWR1-hexasome and SWR1-hexasome-Chz1-dimer complexes

**Figure 6:**
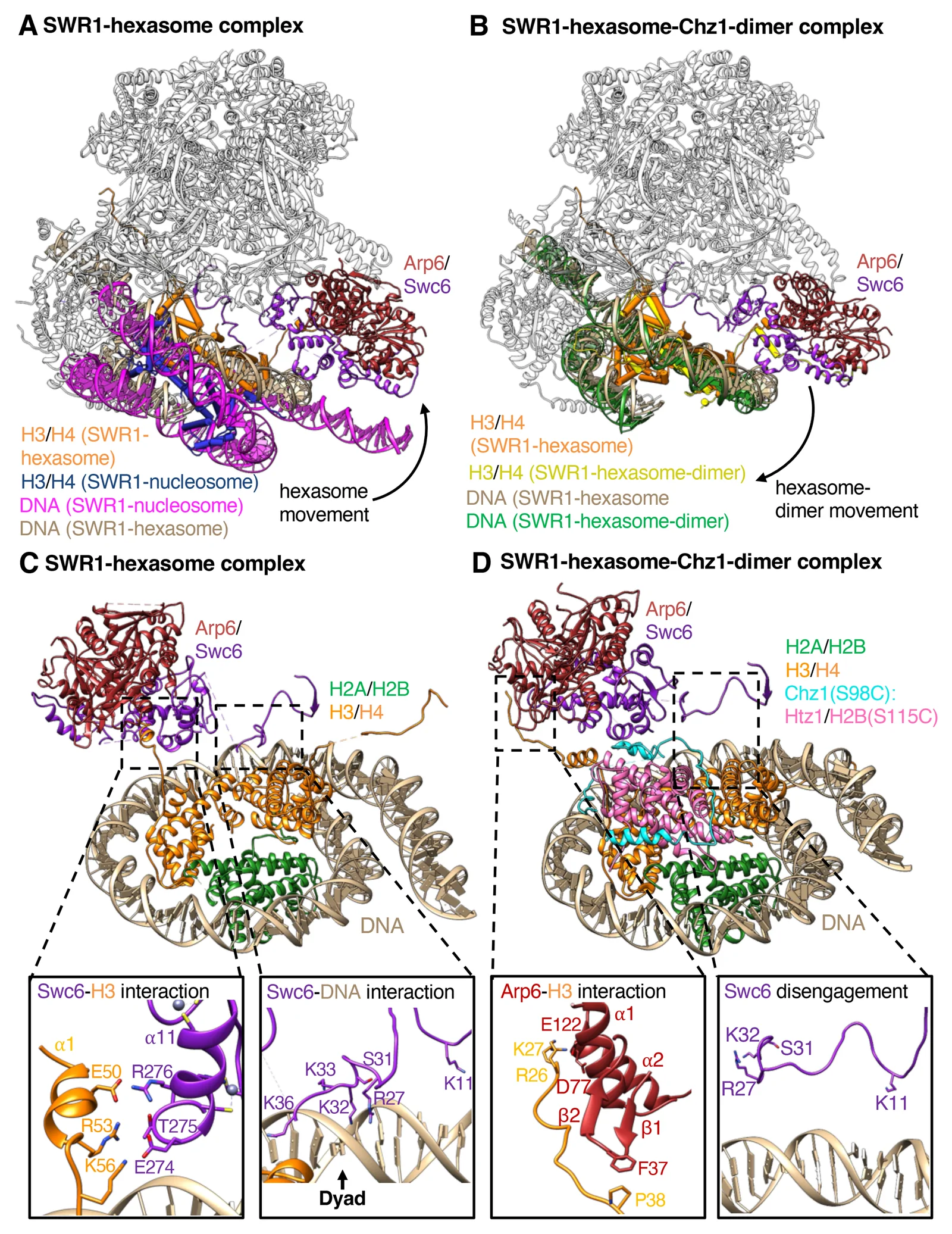
Structural differences between the SWR1-nucleosome, SWR1-hexasome and SWR1-hexasome-Chz1-dimer complexes. **(A)** SWR1 binds the hexasome intermediate more deeply than the nucleosome. The SWR1-nucleosome complex was superimposed onto the SWR1-hexasome complex using the RuvBL1/RuvBL2 heterohexamer as reference. The relative movement of the hexasome towards SWR1 is shown by arrows which show the movement of the lower gyre that remains wrapped around histones in both cases. Much of the upper gyre is unwrapped in the hexasome. For clarity, in the SWR1-hexasome the H2A/H2B histones are omitted, while in the SWR1-nucleosome structure only the DNA and H3/H4 histones are shown. **(B)** SWR1 binds the hexasome intermediate more deeply than the hexasome-Chz1-dimer intermediate. The SWR1-hexasome complex was superimposed onto the SWR1-hexasome-Chz1-dimer complex using the RuvBL1/RuvBL2 heterohexamer as reference. The relative movement of the hexasome-Chz1-dimer is shown by an arrow. For simplicity, in the SWR1-hexasome-Chz1-dimer, the H2A/H2B histones and Chz1(S98C):Htz1/H2B(S115C) were omitted, while in the SWR1-nucleosome structure only the DNA and H3-H4 histones are shown. **(C)** Position of the Arp6/Swc6 complex in the SWR1-hexasome structure. For clarity, only the Arp6/Swc6, histones and DNA in the SWR1-hexasome structure are shown. Close up views detail the interactions between Swc6 and (left) H3 histone (only the zinc-binding fold of Swc6 and the interacting α1 helix of H3 histone are shown), and (right) hexasomal DNA. **(D)** Position of the Arp6/Swc6 complex in the SWR1-hexasome-Chz1-dimer structure. For clarity, only the Arp6/Swc6, Chz1(S98C):Htz1/H2B(S115C), histones and DNA in the SWR1-hexasome-Chz1-dimer structure are shown. Close up views detail the interactions between Arp6 (left) with the H3 histone tail (only α1, α2, β1 and β2 of Arp6 and the H3 histone tail are shown), and Swc6 (right) which disengages from hexasomal DNA.

The overall architecture of the three structures is very similar. The most obvious difference is the unwrapping of the portion of the DNA wrap that curls around the histone dimer in the nucleosome to release the dimer in the hexasome structure (Figures 1E, 6A, S4 & Movies M1-M3). A partial unwrapping takes place in the initial interaction with a nucleosome which is stabilised by interaction with Arp6/Swc6. On progression to the hexasome intermediate, further unwrapping takes place and the DNA is handed off to the Swr1 motor domains and Swc5. Presumably, after dimer replacement, the DNA is then re-wrapped (either actively or simply released) to yield the exchanged nucleosome product.

Another significant difference between the structures is the location of the bound nucleosome or hexasome in relation to the SWR1 complex. The hexasome alone binds into the SWR1 complex more deeply, presumably to protect the exposed surface of the histone core (Figure 6A & Movie M4). When the chaperone-dimer binds, the hexasome lifts slightly to accommodate this extra protein component but is still bound more deeply than the nucleosome (Figure 6B). The binding of the hexasome involves the Arp6/Swc6 module (Figure 6C) that makes extensive interactions across the interface that is exposed by unwrapping of the DNA in the hexasome structures. Such interactions between Swc6 and the hexasomal DNA contributes towards the shift in positions of hexasome substrates relative to the SWR1 complex (Figure 6C). Additionally, in the SWR1-hexasome structure the zinc-binding fold of Swc6 interacts with the surface exposed histone α1 of H3 (Figure 6C & Movie M4), which is then this released when Chz1:Htz1/H2B is deposited, but still remains partially bound to Arp6 (Figure 6D), priming the H3 histone in a position that is compatible with the heterotypic nucleosome.

Taken together, these conformational changes would stabilise the unwrapped state of the nascent (unwrapped) nucleosome prior to displacement of Chz1 suggesting a state which is locked until Chz1 has been displaced.

## Discussion

Previous studies have revealed how SWR1 complex interacts with nucleosomes^8,11^. The ATPase motor domains interact with the DNA wrap at SHL2 but a long section of DNA is unwrapped and stabilised by the Arp6/Swc6 module. The structure was trapped at the initial step of the reaction with an ATP analogue (ADP.BeF3) that locked the complex in a state in which a single base pair had been translocated towards the motor domains. Previous biochemical studies^11,15^ have shown that multiple rounds of ATP hydrolysis drive translocation of the DNA without sliding of the nucleosome. We now show that, on forming the hexasome intermediate in the reaction, a very large region of DNA has become unwrapped and is stabilised away from the nucleosome to allow access to the H2A/H2B or Htz1/H2B dimers. How the complex proceeds from the initial structure to the intermediate remains unclear. Nonetheless, the structures we present here show that the end result is a handoff of the DNA wrap from the Arp6/Swc6 module to Swc5 and the Swr1 subunit. Arp6/Swc6 then plays a different role in stabilising the interface that becomes exposed as a result of unwrapping of the DNA (presumably) by the motor domains. This dual role is shared by the Arp5/Ies6 module of INO80^18–20^. Whether the SC1 component also plays a role in this process is unclear from our structures, in which SC1 remains disordered, but this seems a possibility given this role for SC1 in the related complex INO80^30^.

It has been reported that INO80 complex, a complex that is the archetypal member of the chromatin remodelling enzyme family that includes SWR1^7^, slides hexasomes as well as nucleosome substrates^20,21,30^ and that hexasomes might even be the preferred substrate via a mechanism that involves (temporary) removal or dislodging of a dimer from the histone core^31^. However, although SWR1 is unable to exchange dimers in nucleosomes that have the dimers crosslinked, INO80 can slide crosslinked nucleosomes equally as well as uncrosslinked ones^21^ (Figure S5). Consequently, we conclude that INO80 does not require dimer removal or displacement before then sliding the resulting hexasome, despite being in the same enzyme family as SWR1, even though INO80 can slide hexasomes as well as nucleosomes. Hexasomes and nucleosomes can clearly both be substrates for INO80. However, like many other remodellers, INO80 is not only able to slide nucleosomes but is also able to space them evenly along DNA^32^. By contrast, SWR1 does not slide nucleosomes and a hexasome is an intermediate on the reaction pathway. As observed previously^33,34^, despite being classified as being in the same remodeller family and sharing many subunits in common^7^, SWR1 and INO80 are remarkably different in their activities and interactions with nucleosome substrates.

**Figure 7:**
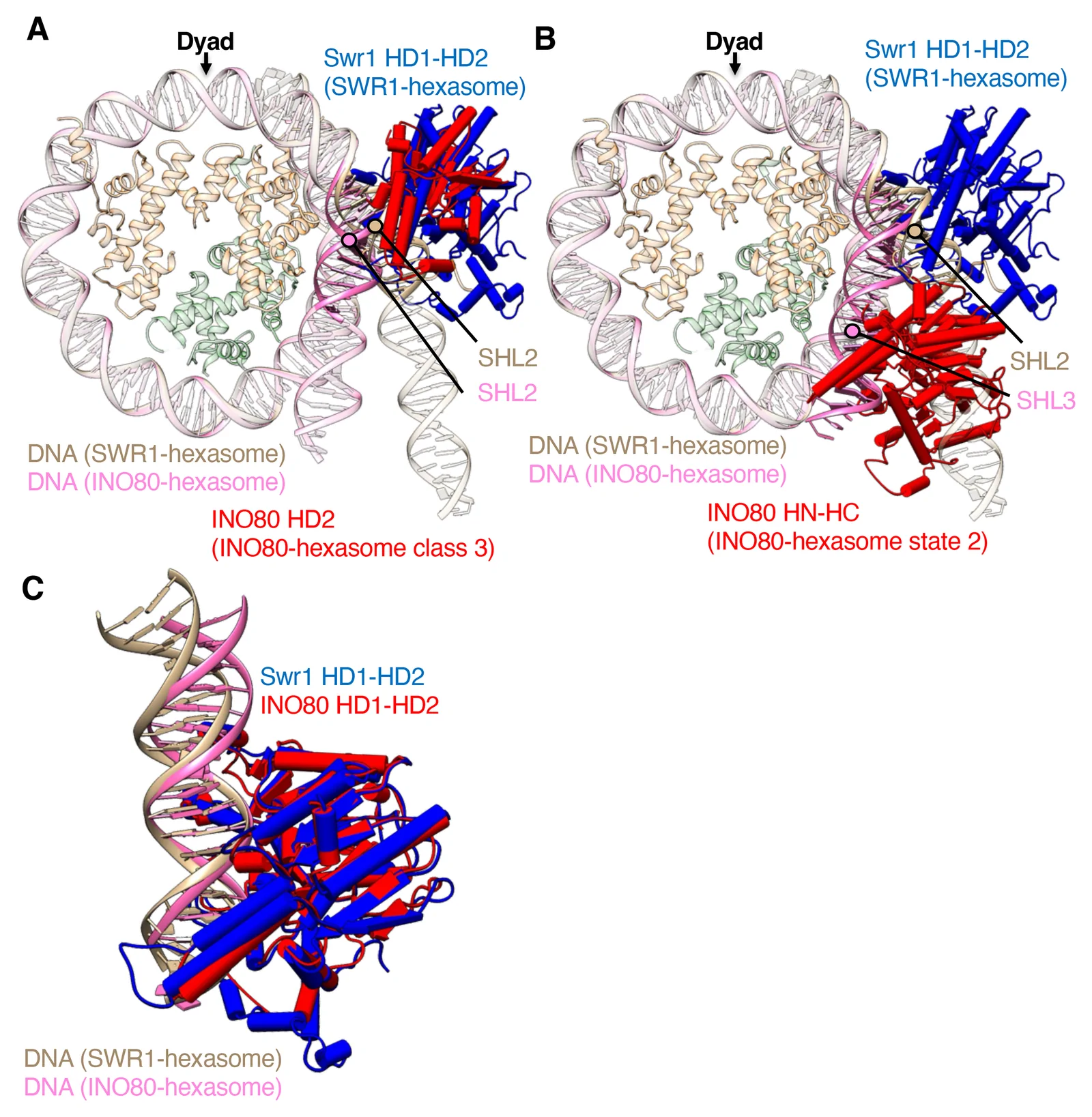
Comparison of engagement of the hexasome by Swr1 and Ino80 motor domains. **(A)** In one observed state of the INO80-hexasome complex, Ino80 (like Swr1) binds at SHL2. For clarity, in the SWR1-hexasome structure only the histones, hexasomal DNA and Swr1 (HD1-HD2, motor domains) are shown, while in the INO80-hexasome structure in class 3 (PDB:8ETW & 8EU2), only the hexasome DNA and the Ino80 HD2 are shown (the HD1 domain of Ino80 is disordered in the structure). **(B)** In another state of the INO80-hexasome complex, the Ino80 motor domains bind at a different position on the hexasome (SHL3). The SWR1-hexasome structure and the INO80-hexasome state 2 structure (PDB:8OOP) were superimposed onto the hexasome core histones. For clarity, in the SWR1-hexasome structure, only the histones, hexasomal DNA and Swr1 HD1-HD2 domains are shown. Similarly, in the INO80-hexasome structure, only the hexasomal DNA and Ino80 HD1-HD2 domains are shown. **(C)** Superimposition of Swr1 HD1-HD2 (SWR1-hexasome) and Ino80 subunit HD1-HD2 (INO80-hexasome state 2 (PDB:8OOP)) highlighting that Swr1 HD1-HD2 and Ino80 HD1-HD2 domains bind to essentially linear DNA unlike that observed for SWR1, and other remodellers bound to nucleosomes at SHL2, where the DNA is bent (Figure 2A).

Indeed, detailed comparison of the SWR1 and INO80 hexasome complexes suggests previous mechanistic comparisons may be simplistic^20,21,30^. INO80 requires dimerization to slide nucleosomes^35^ unlike SWR1 which acts as a monomer for histone exchange^9^. INO80 slides nucleosomes while SWR1 does not. The binding of SWR1 to both nucleosomes and hexasomes is with the motor domains of Swr1 located at SHL2 (ref 8 & this work), the former confirmed by biochemical studies^11^. By contrast, structural data show the INO80 motor domains binding to nucleosomes at SHL6 or SHL7^18,19^, again in agreement with biochemical studies^33^. Reported structures of INO80 bound to a hexasome show a mixture with motor domains bound at SHL2, SHL2.5 and SHL3 with the majority reported at SHL2 (nucleotide free complex^21^) (Figure 7A), or principally at SHL3 (nucleotide bound^20^) (Figures 7B & 7C). Binding at SHL2 is the canonical location for binding of most nucleosome sliders when binding to nucleosomes^36–40^. The exception is INO80, for which biochemical and structural evidence clearly shows the binding site to be at SHL6/7 on nucleosomes^18,19,33^. This difference is important because the DNA is curved at SHL2 as it wraps around the histone core but is linear when unwrapped in the INO80 complex structures both with nucleosomes^18,19^ and hexasomes^20,21^. In fact, the binding of the INO80 motor domains to both hexasome and nucleosome substrates is to unwrapped DNA adjacent to a curved (wrapped) section, in contrast to all other remodeller structures which bind to curved DNA within the wrap at SHL2^8,36–40^, even though many unwrap large segments of DNA from the nucleosome. Furthermore, biochemical evidence suggests INO80 can slide hexasomes (but not nucleosomes) independently of the acidic patch^20^, despite the acidic patch being a common recognition platform recognised, and often required, by many chromatin modifying systems^39,41–44^. In SWR1, by contrast, the Swc2 subunit interacts similarly with the acidic patch of the distal H2A/H2B in both the hexasome and nucleosome bound states^8^. Together, these observations strongly indicate a different mechanism for INO80 and the comparison with other remodellers is misleading even with hexasome substrates.

## Limitations of the study

Although we have now shed some light on how SWR1 stabilises the hexasome intermediate, several important mechanistic questions remain. Not least of all, how DNA is unwrapped during the progression from nucleosome to hexasome remains unclear. Biochemical and structural data suggest there is at least some limited translocation of the DNA wrap^8,11^, it remains unclear whether the unwrapping is progressive or whether some sort of distortion of the DNA is induced that causes spontaneous unwrapping which is then trapped by SWR1. Likewise, the events subsequent to Htz1/H2B dimer insertion remain unclear. The re-wrapping mechanism could be as simple as a release of the constrained DNA to allow a spontaneous re-wrapping. However, a more complex mechanism by which the SWR1 complex replaces the DNA wrap into position cannot be excluded.

## Star★Methods

### Key Resources Table

**Table:** 

REAGENT or RESOURCE	SOURCE	IDENTIFIER
Bacterial and virus strains		
NEB 5-alpha Competent *E. coli*	NEB	C2987H
BL21-CodonPlus-RP	Aglient	50-125-351
Chemicals, peptides, and recombinant proteins		
cOmplete ULTRA tablets,EDTA-free	Roche	5892953001
Benzonase	SIGMA	9025-65-4
Biotin	SIGMA	58-85-5
ATP	SIGMA	34369-07-8
ADP	SIGMA	20398-34-9
NaF	SIGMA	7681-49-4
BeCl	SIGMA	7787-45-5
TCEP	Fluorochem	M02624-25g
DTT	Melford	3483-12-3
2-Mercaptoethanol	SIGMA	60-24-2
BSA	SIGMA	9048-46-8
Ethidium Bromide Solution	SIGMA	1239-45-8
IPTG	SIGMA	367-93-1
Desthiobiotin	IBA	2-1000-002
NaCl_2_	Thermo Fisher Scientific	BP358212
MgCl_2_	VMR Chemicals	0288-500G
Ethyl acetate	SIGMA	141-78-6
HEPES	SIGMA	H 4034-500G
Imidazole	ACROS Organics	122025000
Glycerol	Honeywell	49770-2.5L
SF900 II Serum Free Media	Lonza	Cat# BELN12-730Q
Alexa Fluor 488 C5 Maleimide	Thermo Fisher Scientific	A10254
Alexa Fluor 555 C5 Maleimide	Thermo Fisher Scientific	A20346
Protocatechuic acid	SIGMA	99-50-3
Protocatechuate-3,4-dioxygenase from *Pseudomonas*sp.	SIGMA	P8279
8.0M Guanidine HCl	Thermo Fisher Scientific	10167783
L-glutathione Oxidised	SIGMA	G4376-100G
Trolox	SIGMA	53188-07-1
Critical commercial assays		
5 mL Streptactin Superflow	IBA	2-1240-001
1 mL HiTrap Q HP	Cytiva	GE29-0513-25
1 mL HiTrap Heparin HP	Cytiva	GE17-0407-01
5 mL HiFliQ Ni Adv FPLC	Protein Ark	HiFliQ5-Ni-Adv-1
Superdex 200 Increase 10/300 GL	GE Healthcare	GE28-9909-44
In-Fusion HD Cloning Kit	Takara Bio	639650
GeneJET Plasmid Miniprep Kit	Thermo Scientific	K0503
GeneJET Gel Extraction Kit	Thermo Scientific	K0692
Deposited data		
Map: SWR1-hexasome complex	This study	EMDB: 18764
Map: SWR1(Δswc5)-hexasome complex	This study	EMDB: 50297
Map: SWR1-hexasome-dimer complex	This study	EMDB: 18769
SWR1-hexasome complex	This study	PDB: 8QYV
SWR1(Dswc5)-hexasome complex	This study	PDB: 9FBW
SWR1-hexasome-dimer complex	This study	PDB: 8QZ0
Raw uncropped gel images	This study	Mendeley (DOI : 10.17632/pm4fnm6t 9.-1)
Experimental models: Cell lines		
*Spodoptera frugiperda* Sf9	Thermo Fisher Scientific	11496015
*Trichoplusia ni* High Five	Thermo Fisher Scientific	B85502
Oligonucleotides		
TGCCGTCTGCGAAGGTACTAGGGCTGTTAC (cloning S115C into H2B forward)	IDT	N/A
CCTTCGCAGACGGCATGTTTAGCCAATTCA (cloning S115C into H2B reverse)	IDT	N/A
TGATATTACAGGTATCAATTTCTGCCAAATCGTC (cloning S98C into Chz1 forward)	IDT	N/A
ATACCTGTAATATCATCACATCCGGAAGAAG (cloning S98C into Chz1 reverse)	IDT	N/A
AAGAGGTTGCTACGCCCAAAGAATTGGTTCTGGT (cloning N39C into H2B forward)	IDT	N/A
GCGTAGCAACCTCTTCTTAGCAATCTGTGC (cloning N39C into H2B reverse)	IDT	N/A
CCATCATGCCGAAAGATATTAAACTGGCCCGTCG (cloning Q120M, K121P into H3 forward)	IDT	N/A
CTTTCGGCATGATGGTCACACGTTTCGC (cloning Q120M, K121P into H3 reverse)	IDT	N/A
AGATATTCAGCTGGCCCGTCGTCTGCGT (cloning K125Q in H3 [Q120M, K121P forward])	IDT	N/A
GCCAGCTGAATATCTTTCGGCATGATGGTCACA (cloning K125Q in H3 [Q120M, K121P reverse])	IDT	N/A
Alexa647 - ATCTGGACAATCCCGGTGCCGAGGCCGCTCAATTGGTCGT	IDT	N/A
Cy5-ATCTGGACAATCCCGGTGCCGAGGCCGCTCAATTGGTCGT	IDT	N/A
CTACGACCAATTGAGCGGCCTCGGCACCGGGATTGTCCAGAT (complementary oligo to Alexa647 or Cy5 labelled DNA)	IDT	N/A
Biotin-ATCGGCTGTGTGCACGAACCC	IDT	N/A
GATGTACTCGGGGTGGCGATAAGTCGTGTCTTACC (complementary oligo to Biotin labelled DNA	IDT	N/A
Recombinant DNA		
pFUBB:SWR1(SC1)	This study	N/A
pFUBB:SWR1(SC2)	This study	N/A
pFUBB:SWR1(SC1Dswc5)	This study	N/A
pET22b:Chz1	This study	N/A
pET22b:Chz1(S98C)	This study	N/A
pCDF(NdeI):Htz1/H2B(S115C)	This study	N/A
pETDUET:H3(Q120M,K121P,K125Q)/H4	This study	N/A
pCDF(NdeI):H2A(N39C)/H2B	This study	N/A
Software and algorithms		
cryoSPARC v3.31	Punjani et al ^48^	https://cryosparc.com
RELION 4.0	Scheres et al ^49^	https://www.mrc-lmb.cam.ac.uk/relion/index.php?title=Main_Page
UCSF Chimera	Petterson et al ^50^	https://www.cgl.ucsf.edu/chimera
COOT	Emsley et al ^51^	https://www.mrc-lmb.cam.ac.uk/personal/pemsley/coot/
AlphaFold v2.1.0	Jumper et al ^24^	https://colab.research.google.com/github/deepmind/alphafold/blob/main/notebooks/AlphaFold.ipynb
Image Lab 6.1	BIO RAD	https://www.bio-rad.com/en-uk/product/image-lab-software?ID=KRE6P5E8Z
Model Angelo	Jamali et al ^52^	https://github.com/3dem/model-angelo
RoseTTAFold	Baek et al ^53^	https://robetta.bakerlab.org/
vbFRET	Bronson et al ^54^	https://vbfret.sourceforge.net/
Igor Pro 8	WaveMetrics	https://www.wavemetrics.net/previous8.html
Excel 16.54	Microsoft	https://scicrunch.org/resolver/SCR_016137
Phenix	Liebschner et al ^55^	https://phenix-nline.org/
ChimeraX	Goddard et al ^56^	https://www.rbvi.ucsf.edu/chimerax

**Table:** 

REAGENT or RESOURCE	SOURCE	IDENTIFIER
TGCCGTCTGCGAAGGTACTAGG GCTGTTAC (cloning S115C into H2B forward)	IDT	N/A
CCTTCGCAGACGGCATGTTTAG CCAATTCA (cloning S115C into H2B reverse)	IDT	N/A
TGATATTACAGGTATCAATTTCT GCCAAATCGTC (cloning S98C into Chz1 forward)	IDT	N/A
ATACCTGTAATATCATCACATCC GGAAGAAG (cloning S98C into Chz1 reverse)	IDT	N/A
AAGAGGTTGCTACGCCCAAAGA ATTGGTTCTGGT (cloning N39C into H2B forward)	IDT	N/A
GCGTAGCAACCTCTTCTTAGCAA TCTGTGC (cloning N39C into H2B reverse)	IDT	N/A
CCATCATGCCGAAAGATATTAAA CTGGCCCGTCG (cloning Q120M, K121P into H3 forward)	IDT	N/A
CTTTCGGCATGATGGTCACACG TTTCGC (cloning Q120M, K121P into H3 reverse)	IDT	N/A
AGATATTCAGCTGGCCCGTCGT CTGCGT (cloning K125Q in H3 [Q120M, K121P forward])	IDT	N/A
GCCAGCTGAATATCTTTCGGCAT GATGGTCACA (cloning K125Q in H3 [Q120M, K121P reverse])	IDT	N/A
Alexa647 - ATCTGGACAATCCCGGTGCCGAGGCCGCTCAATTGGTCGT	IDT	N/A
Cy5-ATCTGGACAATCCCGGTGCCGAGGCCGCTCAATTGGTCGT	IDT	N/A
CTACGACCAATTGAGCGGCCTC GGCACCGGGATTGTCCAGAT (complementary oligo to Alexa647 or Cy5 labelled DNA)	IDT	N/A
Biotin-ATCGGCTGTGTGCACGAACCC	IDT	N/A
GATGTACTCGGGGTGGCGATAAGTCGTGTCTTACC (complementary oligo to Biotin labelled DNA	IDT	N/A

## Resource Availability

### Lead contact

Further information and requests for resources and reagents should be directed to and will be fulfilled by the lead contact, Dale B. Wigley (d.wigley@imperial.ac.uk).

### Materials availability

Plasmids generated in this study are available upon request, which should be directed to the lead contact, Dale B. Wigley (d.wigley@imperial.ac.uk).

## Experimental Model And Study Participant Details Method Details

### Bacterial strains

NEB 5-alpha *E. coli* strain was transformed with a pUC18 plasmid containing a multicopy cassette of a Widom 601 fragment and grown overnight at 37°C. BL21-CodonPlus-RP strain (Agilent) *E. coli* strain was transformed with histone expression plasmids and grown at 37°C. Expression was induced by addition of 0.5 mM IPTG and harvested after 3-4 h.

### Insect cells lines

*Spodoptera frugiperda* (Sf9) and *Trichoplusia ni* High Five (BTI-TN-5B1-4, Hi5) insect cells were grown in Insect-XPRESS (Lonza) at 27°C with 140 rpm agitation. Baculovirus generation and amplification was carried out using Sf9 cells. For protein expression Hi5 cells were used, infecting at a cell density of 1.5×10^6^ cells/ml with amplified baculovirus. Hi5 cells were harvested 72 hours after infection.

### Expression and purification of SWR1 and SWR1(Δswc5) complex

Genes encoding for each of the subunits of the SWR1 complex or the SWR1(Δswc5) complex were split across two baculoviruses which were then co-infected to express the full complex in Hi5 cells (**Lin et al., 2017**). For the SWR1(Δswc5) complex, the gene encoding the *swc5* subunit was omitted, as previously reported cells (**Lin et al., 2017**). Cells were lysed by sonication in 50 mM HEPES pH 8.0, 0.5 M NaCl, 1 mM TCEP, 10% glycerol, 1 mM benzamidine-HCL supplemented with 1 protease inhibitor tablet (cOmplete, EDTA-free) and 10 μl of benzonase (Sigma) per liter of cell culture. Lysate was clarified by centrifugation at 30,000 rcf for 60 min at 4°C. The clarified lysate was filtered (through 5 μm and 0.45 μm filters) before being injected onto a StrepTrap HP (Cytiva) column. The column was washed with Buffer A (25 mM HEPES pH 7.5, 0.3 M NaCl, 1mM TCEP, 10% glycerol) before being eluted with Buffer A supplemented with 5 mM desthiobiotin. The eluted protein was combined and diluted 1:1 with Buffer B (25 mM HEPES pH 7.5, 0.1 M NaCl, 1 mM TCEP, 10% glycerol) to dilute the salt before being loaded onto a HiTrap Q HP (Cytiva) column. The protein was eluted with a linear gradient from Buffer B to Buffer C (25 mM HEPES pH 7.5, 2 M NaCl, 1 mM TCEP, 10% glycerol). The relevant fractions were pooled and diluted again 1:1 with Buffer B to reduce the salt before being injected onto a Heparin HP (Cytiva) column. Protein was eluted with a linear gradient from Buffer B to Buffer C. Finally, the protein was concentrated, snap-frozen in liquid nitrogen and stored at - 80°C.

### Expression and purification of human INO80 complex

Genes encoding for each of the subunits of the INO80 complex were split across two baculoviruses which were then co-infected to express the full complex in Hi5 cells. Cells were lysed by sonication in 50 mM HEPES pH 8.0, 0.5 M NaCl, 1 mM TCEP, 10% glycerol, 1 mM benzamidine-HCL supplemented with 1 protease inhibitor tablet (cOmplete, EDTA-free) and 5 μl of benzonase (Sugna) per liter of cell culture. Lysate was clarified by centrifugation at 30,000 rcf for 60 min at 4°C. The clarified lysate was filtered (through 5 μm and 0.45 μm filters) before being injected onto a StrepTrap HP (Cytiva) column. The column was washed with Buffer A (25 mM HEPES pH 7.5, 0.3 M NaCl, 1 mM TCEP, 10% glycerol) before being eluted with Buffer A supplemented with 5 mM desthiobiotin. The eluted protein was combined and diluted 1:1 with Buffer B (25 mM HEPES pH 7.5, 0.1 M NaCl, 1 mM TCEP, 10% glycerol) to dilute the salt before being loaded onto a Heparin HP (Cytiva) column. Protein was eluted with a linear gradient from Buffer B to Buffer C. Finally, the protein was concentrated, snap-frozen in liquid nitrogen and stored at -80°C.

### Expression and purification of Chz1 and Chz1(S98C)

Cell pellets were resuspended in lysis buffer (25 mM HEPES pH 8, 0.5 M NaCl, 1 mM TCEP, 10% glycerol) plus one protease inhibitor tablet and 2 μl benzonase per litre of cell culture and lysed by sonication. Lysate was clarified by centrifugation at 30,000 rcf for 60 min at 4 °C. The clarified lysate was filtered (through 5 μm and 0.45 μm filters) before being loaded onto a cOmplete His-Tag (Roche) purification column. The protein was eluted in lysis buffer supplemented with 250 mM imidazole. Pooled fractions containing Chz1 were diluted with 5x volume of 25 mM HEPES pH 8, 1 mM TCEP, 10% glycerol, to reduce the salt concentration to ~100 mM, before loading on a HiTrap Q HP (Cytiva). Chz1 was eluted using a gradient of 100 mM to 1 M NaCl in 25 mM HEPES pH 8, 1 mM TCEP, 10% glycerol. The same procedure was used to express and purify Chz1(S98C).

### Formation and purification of Chz1:Htz1/H2B complex, covalently linked Chz1(S98C):Htz1-H2B(S115C)

Htz1/H2B dimer and Chz1 or Htz1/H2B(S115C) dimer and Chz1(S98C) were mixed (1.2:1 molar ratio) and unfolded by adding guanidine hydrochloride to a final concentration of 6 M. The mixture was incubated at room temperature for 30 minutes before dialyzing against refolding buffer (25 mM HEPES pH7.5, 150 mM NaCl, with or without 1 mM TCEP) at 4°C. For the covalently linked Chz1(S98C):Htz1/H2B(S115C) the final dialysis buffer also contained 1 mM oxidized glutathione. The refolded Chz1:Htz1-H2B or Chz1(S98C):Htz1/H2B(S115C) complex was then purified either by size exclusion chromatography (Superdex 200 Increase 10/300 GL) or using a heparin column (HiTrap Heparin HP). Complex stoichiometry was assessed by SDS-PAGE and the relevant fractions were combined, snap frozen and stored at –80°C.

### Expression of Saccharomyces cerevisiae histones

*S. cerevisiae* octamers with and without AlexaFluor555 on H2A K119C were expressed in *Escherichia coli* as described (**Lin et al., 2017**). *S. cerevisiae* octamers containing H2A(N39C) were expressed in *E. coli* in the same way, but with omission of reducing agent. To ensure complete formation of a disulfide bond between each H2A(N39C) within the octamer, the octamer was dialyzed against 20 mM Tris pH 8.8, 1 mM EDTA, 2 M NaCl and 1 mM oxidized glutathione. (**Frouws, 2018**).

*S. cerevisiae* H2A/H2B, Htz1/H2B (with or without AlexaFluor 555 on Htz1 K125C) and Htz1/H2B(S115C) histone dimers were expressed in *E. coli* and purified as soluble dimers. Cells were lysed by sonication in Buffer D (20 mM Tris, pH7.5, 0.5 M NaCl, 0.1 mM EDTA, 1 mM TCEP) plus protease inhibitor tablets (Roche, 2 tablets per 100 ml). Dimers were purified by loading the cleared lysate onto tandem HiTrap Q FF and HiTrap Heparin HP columns in Buffer E (20 mM Tris, pH7.5, 0.5 M NaCl, 1 mM EDTA, 1 mM TCEP). The HiTrap Q FF column was removed prior to elution from the HiTrap Heparin HP column via a gradient to Buffer F (20 mM Tris, pH7.5, 2 M NaCl, 1 mM EDTA, 1 mM TCEP), followed by gel filtration on a Superdex S200 in Buffer F.

*S. cerevisiae* histone H3(Q120M, K121P, K125Q) and histone H4 were co-expressed in *E. coli and purified as soluble tetramers. Cells were lysed by sonication in* Buffer D plus protease inhibitor tablets (Roche, 2 tablets per 100 ml). Tetramers were purified using a HiTrap Heparin HP column in Buffer E and eluted via a gradient to Buffer F, followed by gel filtration on a Superdex S200 in Buffer F.

### Preparation of nucleosomes

DNA containing the Widom 601 sequence was generated as described (**Willhoft et al., 2018**). Salt gradient dialysis of the *S. cerevisiae* octamers with DNA was carried out to form a ‘core’ nucleosome. A DNA overhang was ligated to the core nucleosome as described (**Willhoft et al., 2018**). Following ligation, the final nucleosomes were either 62N2 or 113N2. For nucleosomes that were used in single-molecule experiments a biotinylated overhang was used, resulting in ^biotin^113N2. For nucleosomes where the DNA was labeled (with either AF647 or Cy5), the fluorophore was attached at the end of the 2 bp overhang as described (**Willhoft et al., 2018**). Nucleosomes containing a disulfide bond between the two H2A(N39C) histones were prepared in the same way, but with the omission of reducing agent.

### Preparation of hexasomes

To facilitate the formation of yeast hexasomes, three amino acid substitutions were introduced into the *S. cerevisiae* H3 histone (Q120M, K121P, K125Q) (**Leung et al., 2016**).

To form hexasomes, *S. cerevisiae* H2A/H2B dimers were mixed with *S. cerevisiae* H3^MPQ^-H4 tetramers (at a 0.6:1 ratio to ensure only partial H2A/H2B occupancy). Hexasomes were assembled onto the same DNA that was used for nucleosomes by salt gradient dialysis to generate ‘core’ hexasomes. Core hexasomes were separated from tetrasomes, nucleosomes and free DNA using a MonoQ column, loaded in buffer G (20 mM Tris, pH 7.5, 1 mM EDTA, 1 mM TCEP, 200 mM NaCl) eluting with a gradient into buffer H (as buffer G with 2M NaCl). The fractions were immediately diluted into 4x volume of 20 mM Tris pH 7.5 to reduce the salt concentration. A DNA overhang was ligated to the core hexasome in the same way as was used for nucleosomes, resulting in 113H2 or ^biotin^113H2 hexasomes (‘H’ representing a hexasome assembled on the Widom 601 sequence). For hexasomes where the DNA was labeled, the fluorophore was attached at the end of the 2 bp short overhang.

As is the case for hexasomes prepared with *X. laevis* histones (**Levendosky et al., 2016**), yeast hexasomes prepared in this way exploit the inherent asymmetry of the Widom 601 sequence. Because of this asymmetry, the H2A/H2B dimer present in a hexasome is preferentially located on the ‘TA rich’ side of the Widom 601 sequence, leaving the vacant site on the ‘TA poor’ side. We orientate our Widom 601 sequence with the ‘TA rich’ side closest to the 2 bp short overhang. This results in the vacant H2A/H2B site being located next to the 113 bp linker.

### Histone exchange activity measurements of SWR1

Histone exchange activity was measured using either a gel-based assay or a FRET-based microtiter format assay as previously described (**Lin et al., 2017**). For gel-based assays, nucleosomes labeled with AF647 on the 5’ end of the short linker were used. Where stated, 50 nM SWR1 was premixed with 400 nM of either a canonical nucleosome or nucleosome H2A(N39C) and 800 nM of Htz1(K125C) labeled with AF555 /H2B in a reaction buffer [25 mM HEPES pH 7.5 and 50 mM NaCl2) for 120 min at 30°C. Reactions were started by the addition of 1 mM ATP and 2 mM MgCL2 and terminated by the addition of 10% (v/v) glyerol, 10 mM EDTA, 1 μg salmon sperm DNA. Reaction products were resolved by native gel electrophoresis using 6% acrylamide–TBE gels, run in 0.5× TBE buffer at 90 V for 90 min at 4°C. Gels were visualized using a Bio-Rad ChemiDoc MP system.

For FRET-based microtiter assays, nucleosomes labeled on H2A K119C with AF488 and AF647 on the 5’ end of the short linker. Where stated, 50 nM SWR1 was premixed with 400 nM Chz1-Htz1-H2B in the presence of either 200 nM of nucleosome or 200 nM nucleosome (H3^MPQ^). In experiments comparing the histone exchange activity between Chz1:Htz1/H2B and Chz1(S98C)-Htz1-H2B(S115C), 50 nM SWR1 was pre-mixed with either 400 nM Chz1-Htz1-H2B or 400 nM Chz1(S98C)-Htz1-H2B(S115C) or 400 nM Chz1(S98C)-Htz1-H2B pre-incubated with 2 mM DTT for 60 min, before being mixed with 200 nM labelled nucleosomes.

### Nucleosome sliding activity measurement of INO80

Nucleosome sliding activity was measured using a gel-based assay as previously described (**Willhoft et al., 2018**), using nucleosomes labeled with AF647 on the 5’ end of the short linker. Where stated, 100 nM human INO80 was pre-mixed with either 400 nM of either a canonical nucleosome or nucleosome H2A(N39C) for 20 min at 37°C. Reactions were started by the addition of 1 mM ATP and 2 mM MgCL2 and terminated by the addition of 10% (v/v) glycerol, 10 mM EDTA and 1 μg salmon sperm DNA. Reaction products were resolved by native gel electrophoresis using 6% acrylamide–TBE gels, run in 0.5× TBE buffer at 90 V for 90 min at 4°C. Gels were visualized using a Bio-Rad ChemiDoc MP system.

### Preparation of SWR1-hexasome, SWR1(Δswc5)-hexasome, and SWR1-hexasome-dimer complexes for cryoEM

For the SWR1-hexasome complex, SWR1 was premixed with hexasome in the presence of 1 mM ADP:BeF, at a ratio of 1:1 in a cryoEM buffer consisting of buffer [25 mM HEPES pH 7.5, 50 mM NaCl]. Samples were left to incubate at 30°C for 10 mins before being loaded onto a glycerol gradient, in a buffer consisting of [25 mM HEPES pH 7.5, 50 mM NaCl, glycerol 15-45%]. The samples were centrifuged at 22,700 RPM for 16 hours at 4°C. Samples were then fractionated and analyzed using SDS-PAGE. Samples containing all subunits of the SWR1-hexasome complex in stoichiometry were pooled, concentrated and washed 3x in the cryoEM buffer to remove the remaining glycerol in the buffer. Finally, ADP:BeF at a final concentration of 1 mM was added to the concentrated samples. The final concentration of SWR1-hexasome complex used in grid preparation was ~420 nM. The same protocol was used to generate the SWR1(Δswc5)-hexasome complex, with the final concentration of SWR1(Δswc5)-hexasome complex used in grid preparation being ~420 nM. A similar protocol was used to generate the SWR1-hexasome-dimer except SWR1 was premixed with Chz1(S98C):Htz1/H2B(S115C) and the hexasome at a ratio of 1:2:1. The final concentration of SWR1-hexasome-dimer complex used in grid preparation was ~300 nM.

### Grid preparation

Quantifoil™ R2/1 300 copper mesh grids or R2/2 copper mesh grids (Quantifoil) were used in all Cryo-EM experiments. For the SWR1-hexasome complex, Quantifoil™ R2/1 300 copper mesh grids were used, whereas for the SWR1(Δswc5)-hexasome and SWR1-hexasome dimer complexes, Quantifoil™ R2/2 300 (Quantifoil) copper mesh grids were used. Briefly, every grid was cleaned by multiple rounds of washing with water followed by ethyl acetate. Grids were flash-frozen with the aid of an FEI Vitrobot Mark IV (Thermo Fisher Scientific). 4 μL of either the SWR1-hexasome or SWR1-hexasome-dimer sample were deposited onto the grid, followed by a 30 second wait time and 1 second blot time, before being plunged into liquid ethane. The Vitrobot chamber was maintained at close to 100% humidity and at 4°C.

### CryoEM data processing

Motion correction of Movie frames, CTF parameters, and particle picking were performed as previously described (Willhoft et al., 2018). Global and local resolution estimates were calculated based on the gold-standard Fourier shell correlation (FSC = 0.143) criterion.

The cryoEM processing workflow for the 3.5 Å SWR1-hexasome volume is summarized in Figure S2A. 2D classification in cryoSPARC was conducted, selecting for 2D classes containing density for SWR1 resulted in a working particle pool of 517,474 particles (Punjani et al., 2017). These were sub-divided into three classes via heterogeneous refinement in cryoSPARC, resulting in class #1: SWR1-hexasome complex (~18%), class #2: SWR1-apo class (~34%) and class #3: RuvBL1-RuvBL2 only class (~48%) (Punjani et al., 2017). Particles in class #1 were then imported into RELION for one round of 2D classification, resulting in a working pool of 87,671 particles (Scheres, 2012). The selected particles were subjected to 3D refinement in RELION with a mask corresponding to the SWR1 sub-complex of Swr1, Arp6, Swc6, Swc2, Swc5, RuvBL1, RuvBL2, and the hexasome to generate an initial 3.8 Å SWR1-hexasome volume (Scheres, 2012). The generated 3.8 Å SWR1-hexasome volume was then subjected to one round of 3D classification without alignment (T=30) with a soft mask overlapping the H4 histone tail and Swc5, generating class #1.1 (~61%): clear density of H4 and Swc5 and class #1.2 (~34%): lack of density for H4 and Swc5 (Scheres, 2012). Particles in class #1.1 were selected for 3D refinement in RELION with a mask corresponding to the SWR1 sub-complex of Swr1, Arp6, Swc6, Swc2, Swc5, RuvBL1, RuvBL2, and the hexasome. A second round of 3D without alignment (T=30) was done with a soft mask overlapping Arp6-Swc6 and a portion of the nucleosome (Scheres, 2012). This generated two classes, class #1.1.1 (~56%): clear density for the Arp6-Swc6 and class #1.1.2 (~34%) lack of density for the Arp6-Swc6. Particles in class #1.1.1 were selected for 3D refinement in RELION with a mask corresponding to the entire SWR1-hexasome complex, generating an intermediate 3.7 Å SWR1-hexasome volume. These particles were then subjected to non-uniform refinement in CryoSPARC, before a final 3D refinement in RELION with a mask corresponding to the SWR1 sub-complex of Swr1, Arp6, Swc6, Swc2, Swc5, RuvBL1, RuvBL2, and the hexasome to generate the final 3.5 Å SWR1-hexasome map used for model building (Scheres, 2012).

The cryoEM processing workflow for the 4.4 Å SWR1(Δswc5)-hexasome volume is summarized in Figure S2B. 2D classification in cryoSPARC was conducted, selecting for 2D classes containing density for SWR1 resulted in a working particle pool of 481,593 particles (Punjani et al., 2017). These were sub-divided into three classes via heterogeneous refinement in cryoSPARC, resulting in class #1: SWR1(Δswc5)-hexasome complex (~16%), class #2: SWR1-apo class (~46%) and class #3: RuvBL1-RuvBL2 only class (~38%) (Punjani et al., 2017). Particles in class #1 were then imported into RELION for one round of 2D classification, resulting in a working pool of 77,029 particles (Scheres, 2012). The imported particles were then subjected to 3D classification without alignment (T=20) in RELION with a soft mask overlapping the entire SWR1(Δswc5)-hexasome volume, generating class #1.1 (~25%): lack of density for the Swr1 insert domain and certain regions of the Swr1 motor and class #1.2 (~75%): clear density for the Swr1 insert domain and certain regions of the Swr1 motor. Particles in class #1.2 were selected, and subjected to a final 3D refinement in RELION with a tight mask corresponding to the entire SWR1(Δswc5)-hexasome complex, generating the final 4.4 Å SWR1(Δswc5)-hexasome map used for model building.

The cryoEM processing workflow for the 3.8 Å SWR1-hexasome-dimer map is summarized in Figure S2C. 2D classification in cryoSPARC for 2D classes containing density for SWR1 resulted in a working particle pool of 475,531 particles (Punjani et al., 2017). These were sub-divided into three classes via heterogeneous refinement in cryoSPARC, resulting in class #1: SWR1-CZBX-hexasome complex (~20%), class #2: SWR1-apo class (~68%) and class #3: RuvBL1/RuvBL2 only class (~12%). The particles in class #1 were then imported into RELION, and an initial 3D volume was generated using RELION initial model (Scheres, 2012). The generated 3.8 Å SWR1-CZBX-hexasome initial volume was then subjected to one round of 3D classification without alignment (T=30) in RELION with a mask corresponding to the entire SWR1-CZBX-hexasome complex. This generated two classes of particles, class #1.1 (~36%) 33,726 particles and class #1.2 (~64%) (Scheres, 2012). Particles in class #1.1 were selected for 3D refinement in RELION with a mask corresponding to the entire SWR1-hexasome-dimer complex, generating a 3.6 Å intermediate SWR1-hexasome-dimer volume. This intermediate SWR1-CZBX-hexasome volume was then subjected to a second round of 3D classification without alignment (T=30), using a soft mask corresponding to Arp6-Swc6, Chz1(S98C)-Htz1-H2B(S115C), Swr1 insert and Swc5 (Scheres, 2012). This generated two classes of particles, class #1.1.1 (~70%): with clear density for the Arp6/Swc6, Chz1(S98C):Htz1/H2B(S115C), Swr1 insert and Swc5, and class #1.2 (~30%). Particles in class #1.1.1 were selected for a final 3D refinement in RELION with a mask corresponding to the SWR1 sub-complex of Swr1, Arp6, Swc6, Swc2, Swc5, RuvBL1, RuvBL2, Chz1(S98C):Htz1/H2B(S115C) and the hexasome to generate the final 3.8 Å SWR1-hexasome map used for model building.

### Model Building

For the 3.5 Å SWR1-hexasome complex, first the built-in coordinates for the RuvBL1-RuvBL2 hexamer in the previously deposited 3.6 Å SWR1-nucleosome complex (PDB: 6GEJ) were docked into the 3.5 Å SWR1-hexasome volume (Willhoft et al., 2018) using UCSF Chimera (Pettersen et al., 2004). Additional coordinates for one RuvBL2 subunit, whereby density was not previously observed in the 3.6 Å SWR1-nucleosome volume (EMD-4395) was manually built in COOT (Emsley et al., 2004). This was then followed by the built-in coordinates Swr1 motor domain in the previously deposited 3.6 Å SWR1-nucleosome complex (PDB: 6GEJ) (Willhoft et al., 2018), with the Swr1 ATPase domains and the Swr1 insert domain docked in separately using UCSF Chimera (Pettersen et al., 2004). Additional coordinates, whereby density was not previously observed in the 3.6 Å SWR1-nucleosome volume (EMD-4395), as well as differences in conformation of the Swr1 subunit was manually built in COOT (Emsley et al., 2004). The built-in coordinates for the Arp6 subunit, followed by Swc6 subunit in the previously deposited 3.6 Å SWR1-nucleosome complex (PDB: 6GEJ) was then docked into the 3.5 Å SWR1-hexasome volume using UCSF Chimera (Pettersen et al., 2004), with additional residues not previously observed in the 3.6 Å SWR1-nucleosome volume (EMD-4395) built in manually in COOT (Emsley et al., 2004).

Next, in the SWR1-bound nucleosome (PDB: 6GEJ), the chains representing the “evicted” H2A-H2B dimer in nucleosome of the SWR1-bound nucleosome (PDB: 6GEJ) was omitted. The built-in coordinates for the SWR1-bound nucleosome lacking the “evicted” H2A-H2B dimer was then docked into the 3.5 Å SWR1-hexasome volume (Willhoft et al., 2018). Mutations in both copies of the H3 histone of the “hexasome” to alter the sequence to H3^MPQ^ was done in COOT (Emsley et al., 2004). Nucleosomal DNA from the SWR1-bound nucleosome (PDB: 6GEJ), that did not fit the density of the 3.5 Å SWR1-hexasome volume was deleted using UCSF Chimera (Pettersen et al., 2004). The DNA at position super helical location (SHL2) was then manually built in COOT (Emsley et al., 2004). Additional features and differences observed in the 3.5 Å SWR1-hexasome volume, such as the a1 H3 histone and the visible H4 histone tail was manually built in COOT (Emsley et al., 2004). For the Swc2 subunit, an initial template was generated using AlphaFold (Jumper et al., 2021). Different regions corresponding to secondary structures of the AlphaFold generated template was manually truncated and, docked separately into the 3.5 Å SWR1-hexasome volume using UCSF Chimera and COOT (Pettersen et al., 2004, Emsley et al., 2004). To identify the Swc5 subunit, an initial model of Swc5 was generated in AlphaFold, with the final α6-α8 residues fitting well into the additional density. This was further validated using ModelAngelo (Jamali et al., 2023), which generated an initial model that matched the secondary structure of α6-α8 of Swc5 generated via AlphaFold. The built-in residues generated by ModelAngelo were then subjected to protein-BLAST analysis, which demonstrated a high sequence identity to the Swc5 subunit. This led us to assign this additional density as the Swc5 subunit. To build in the Swc5 subunit, an initial model was generated using both AlphaFold (Jumper et al., 2021) and RoseTTAFold (Baek et al., 2021). Different regions corresponding to secondary structures of the generated Swc5 template were manually truncated and docked separately into the 3.5 Å SWR1-hexasome volume using UCSF Chimera and COOT (Pettersen et al., 2004, Emsley et al., 2004). The final built in coordinates of the SWR1-hexasome was subjected to real space refinement in Phenix (Afonine et al., 2018).

For the 4.4 Å SWR1(Δswc5)-hexasome complex, first the built-in coordinates for the RuvBL1-RuvBL2 in the recently built 3.5 Å SWR1-hexasome complex was docked into the 4.4 Å SWR1(Δswc5)-hexasome volume in UCSF Chimera (Pettersen et al., 2004). This was then followed by the coordinates of the Arp6-Swc6 complex in the recently built 3.5 Å SWR1-hexasome complex. The Swc2 subunit was then docked into the 4.4 Å SWR1(Δswc5)- hexasome volume. Different regions corresponding to secondary structures of the generated Swc2 template were manually truncated and docked separately into the 4.4 Å SWR1(Δswc5)- hexasome volume using UCSF Chimera and COOT. Next, the Swr1 motor domain from the 3.6 Å SWR1-nucleosome complex (PDB: 6GEJ) was docked into the 4.4 Å SWR1(Δswc5)- hexasome volume in UCSF Chimera (Pettersen et al., 2004), with the ATPase domains and the Swr1 insert domain docked in and built separately using UCSF Chimera and COOT (Pettersen et al., 2004, Emsley et al., 2004). The hexasome from the 3.5 Å SWR1-hexasome complex was the docked into the 4.4 Å SWR1(Δswc5)-hexasome volume in UCSF Chimera (Pettersen et al., 2004), with different regions being manually built in COOT. The unwrapped DNA was manually built in COOT (Emsley et al., 2004). The final built in coordinates of the SWR1-hexasome was subjected to real space refinement in Phenix (Afonine et al., 2018).

For the 3.8 Å SWR1-hexasome-dimer complex, first the built-in coordinates for the RuvBL1-RuvBL2 in the recently built 3.5 Å SWR1-hexasome complex was docked into the 3.8 Å SWR1-hexasome-dimer volume in UCSF Chimera (Pettersen et al., 2004, Emsley et al., 2004). This was then followed by the coordinates of the Swr1 motor domain in the recently built SWR1-hexasome structure, with the Swr1 ATPase domains and the Swr1 insert domain docked in and built separately using UCSF Chimera (Pettersen et al., 2004). AlphaFold (Jumper et al., 2021) and RoseTTAFold (Baek et al., 2021) model was used to generate the additional residues of the Swr1 ATPase observed in the 3.8 Å SWR1-hexasome-dimer volume. Different regions corresponding to secondary structures of the generated Swr1 template were manually truncated and docked separately into the 3.8 Å SWR1-hexasome volume using COOT and UCSF Chimera (Pettersen et al., 2004, Emsley et al., 2004). The built-in coordinates for the Arp6 subunit, followed by Swc6 subunit in the recently built 3.5 Å SWR1-hexasome complex using UCSF Chimera (Pettersen et al., 2004), with additional residues built in manually in COOT (Emsley et al., 2004). Next, the built-in coordinates for the recently generated SWR1-bound hexasome along with the recently built Swc2 in the SWR1-hexasome structure was docked as a single complex into the 3.8 Å SWR1-hexasome-dimer volume using UCSF Chimera (Pettersen et al., 2004) with additional residues built in manually in COOT (Emsley et al., 2004). Both the coordinates for the NMR structure of the Chz1:Htz1/H2B complex (PDB: 2JSS) and the crystal structure of Chz1:Htz1/H2B complex (PDB:6AE8) was used as a reference for the Chz1(S98C):Htz1/H2B(S115C). AlphaFold multimer was then used to generate a Chz1(S98C):Htz1/H2B(S115C) model that was docked into the 3.8 Å SWR1-hexasome-dimer volume using Chimera (Zhou et al., 2008). Mutations in the Chz1:Htz1/H2B were conducted in COOT (Emsley et al., 2004) to change the sequence to the Chz1(S98C):Htz1/H2B(S115C). Finally, the recently built Swc5 subunit was docked into the 3.8 Å SWR1-hexasome-dimer volume. Different regions corresponding to secondary structures of the template were manually truncated and docked separately into the 3.8 Å SWR1-hexasome-dimer volume. The final built in coordinates of the SWR1-hexasome-dimer was subjected to real space refinement in Phenix (Afonine et al., 2018).

### Single-molecule FRET (smFRET) microscope setup

Single-molecule FRET measurements were performed on a homebuilt prism-TIRF (Total Internal Reflection Fluorescence) microscope. Fluorophores were excited with either a 532 nm laser (Stradus, Vortran) or a 637 nm laser (Stradus, Vortran). Fluorescence was collected through a 1.2 NA, 60x water objective (Olympus) and filtered through a dual bandpass filter (FF01-577/690-25, Semrock). The fluorescence was spectrally filtered using an OptoSplit II (Cairn Research) to separate donor and acceptor emission. The donor and acceptor emissions were further filtered through ET585/65M and ET700/75M (Chroma) bandpass filters, respectively. The donor and acceptor images were then projected side-by-side onto an EMCCD (Andor iXon Ultra 897). Data was collected as raw movies using a custom LabVIEW script at 100 ms time resolution.

Single molecule fluorescence spots from the raw movies were localised using custom IDL scripts and converted into raw fluorescence trajectories. Raw fluorescence trajectories were corrected for bleed-through of the donor fluorescence into the acceptor channel. Apparent FRET efficiencies were calculated as the ratio of acceptor intensity divided by the sum of the donor and acceptor intensities.

### smFRET microscope slide passivation and flow chamber assembly

Quartz slides (UQC optics) and glass coverslips were aminosilinized with N-(2-Aminoethyl)-3-aminopropyltrimethoxysilane then pegylated using methoxy-PEG-SVA (Mr = 5,000, Laysan Bio, Inc.) containing 5 % biotin-PEG-SVA (Mr = 5,000, Laysan Bio, Inc.) in 100 mM sodium bicarbonate as described (**Brenlla et al., 2014**) with minor modifications. Following passivation, slides and coverslips were stored under nitrogen in the dark at -20 °C. Prior to use, slides and coverslips were warmed to room temperature and assembled into flow chambers using 0.12 mm thick double-sided adhesive sheets (Grace Bio-Labs SecureSeal). Flow chambers were sealed with epoxy glue.

### smFRET exchange assay with covalently linked Chz1:Htz1/H2B

Nucleosomes, (^biotin^113N2^Cy5^, H2A^555^) labeled with a FRET donor (AlexaFluor555) on H2A and acceptor (Cy5) on the short end of the DNA overhang were surface-immobilized in a microscope flow chamber assembled as described above. SWR1 (40 nM) with covalently linked Chz1:Htz1/H2B (80 nM) in imaging buffer (25 mM Tris-HCl, pH 7.8, 100 mM KCl, 4% glycerol, 1 mM EDTA, 2 mM MgCl2, 0.2 mg/ml BSA, 17 μM biotin, Trolox, 2.5 mM protocatechuic and 0.25 μM protocatechuate-3,4-dioxygenase) were injected into the flow chamber and the sample imaged. Imaging was carried out as follows, first the acceptor was imaged by direct excitation using the 637 nm laser (for 10-15 s), before switching to the 532 nm laser to image both the FRET donor and acceptor.

To start the exchange reaction, SWR1 (40 nM), covalently linked Chz1:Htz1/H2B (80 nM) and ATP (1 mM) in imaging buffer were injected into the flow chamber. Imaging of the sample was carried out at defined intervals as indicated. At each time point the ratio of FRETing nucleosomes to nucleosomes containing a Cy5 acceptor (as determined by direct Cy5 excitation) was used as a readout for histone exchange. For experiments that used reduced covalently linked Chz1:Htz1/H2B, the chaperone bound Htz1/H2B dimer was first reduced by incubation with 1 mM TCEP prior to the experiment. The experiment was carried out in the same way; with the imaging buffer was supplemented with 1 mM TCEP to maintain reducing conditions.

### Single-molecule assay to monitor insertion of covalently linked Chz1:Htz1/H2B into hexasomes

Hexasomes, labeled with AlexaFluor647 at the end of the short DNA overhang (^biotin^113H2^647^) were surface immobilized in an assembled microscope flow chamber. Covalently linked Chz1:Htz1^555^/H2B (5 nM) with or without SWR1 (5 nM) in imaging buffer (25 mM Tris-HCl, pH 7.8, 100 mM KCl, 4% glycerol, 1 mM EDTA, 2 mM MgCl2, 0.2 mg/ml BSA, 17 μM biotin, Trolox, 2.5 mM protocatechuic and 0.25 μM protocatechuate-3,4-dioxygenase) was injected into the flow cell and imaged. Imaging was carried out as follows, first hexasomes were localized by direct excitation of AlexaFluor647 using the 637 nm laser, before switching to the 532 nm laser to excite AlexaFluor555 on Chz1:Htz1^555^/H2B.

Single-molecule trajectories were inspected manually using custom MATLAB scripts. Chz1:Htz1^555^/H2B binding to a hexasome (without insertion) was observed as an increase in donor intensity without any increase in FRET. Chz1:Htz1^555^/H2B insertion was observed as an increase in donor intensity that also led to an increase in FRET. For plotting FRET histograms, the trace was cropped to just the region displaying FRET. Cropped FRET traces were idealized using vbFRET (**Bronson et al., 2009**) by inferring a Hidden Markov Model. The idealized FRET states were used to generate FRET histograms, plotted using Igor Pro 8 (Wavemetrics).

### Quantification And Statistical Analysis

For the single-molecule assay that monitored insertion of covalently linked Chz1:Htz1/H2B into hexasomes statistical analysis was performed using Bayesian inference. Experiments were performed at least twice and the sum of all events from all repeats was used. A uniform prior distribution, Beta(1,1) was used, such that for n total hexasomes observed where x hexasomes showed insertion of Chz1:Htz1/H2B, α=1+x and β=1+n-x. Therefore, the mean and variance of x are given as,

$$\text{E}[X]=\frac{\alpha }{\alpha +\beta }\;\text{and}\;\mathrm{var}[X]=\frac{\alpha \beta }{{\left(\alpha +\beta \right)}^{2}(\alpha +\beta +1)}$$

Data is reported as mean ± standard deviation.

## Supplementary Material

Supplementary Materials

## Data Availability

The Cryo-EM structures of SWR1-hexasome complex, SWR1(Δswc5)-hexasome complex and SWR1-hexasome-dimer complex have been deposited in the PDB with the accession codes PDB: 8QYV, 9FBW and 8QZ0 respectively. The cryo-EM volumes generated for the SWR1-hexasome complex, SWR1(Δswc5)-hexasome complex and SWR1-hexasome-dimer complex have been deposited in the Electron Microscopy Data Bank with the accession codes 18764, 50297 and 18769 respectively. All uncropped raw gel images have been deposited at Mendeley and are publicly available as of the date of publication (DOI : 10.17632/pm4fnm6t9j.1) This paper does not report original code Any additional information required to reanalyze the data reported in this paper is available from the lead contact upon request

## Data and material availability

Electron density maps are deposited at the Electron Microscopy Database (accession codes EMDB-18764, EMDB-18769 & EMDB-50297) and protein coordinates are deposited at the Protein Databank (PDB ID codes 8QYV, 8QZ0 & 9FBW).
