# Supplementary material for: Stabilisation of the hexasome intermediate during histone exchange by yeast SWR1 complex
Source: Mol Cell. Author manuscript; Available in PMC 2026 Aug 28. (PMC7619409; doi:10.1016/j.molcel.2024.08.015)
Supplement: Supplementary file 1 [file EMS217836-supplement-Supplementary_Materials.pdf]

Movie Legends

Movie M1. Rotating overview of SWR1-hexasome complex coordinates (coloured as in Figure 1C) fitted into the 3.5Å cryo-EM envelope, related to Figure 1 & 3.

Movie M2. Morph between the SWR1-bound nucleosome (PDB:6GEJ) and the SWR1-bound hexasome, which highlights the distortions introduced into the hexasome by SWR1, related to Figure 1.

Movie M3. Rotating overview of SWR1-hexasome-Chz1-dimer complex coordinates (coloured as in Figure 5A) fitted into the 3.8Å cryo-EM envelope, related to Figure 5.

Movie M4. Morph between SWR1-nucleosome complex (PDB:8QKU) and the SWR1-hexasome complex, which highlights the shift in position of the hexasome relative to the SWR1 main body, related to Figure 6.

FIGURE S1

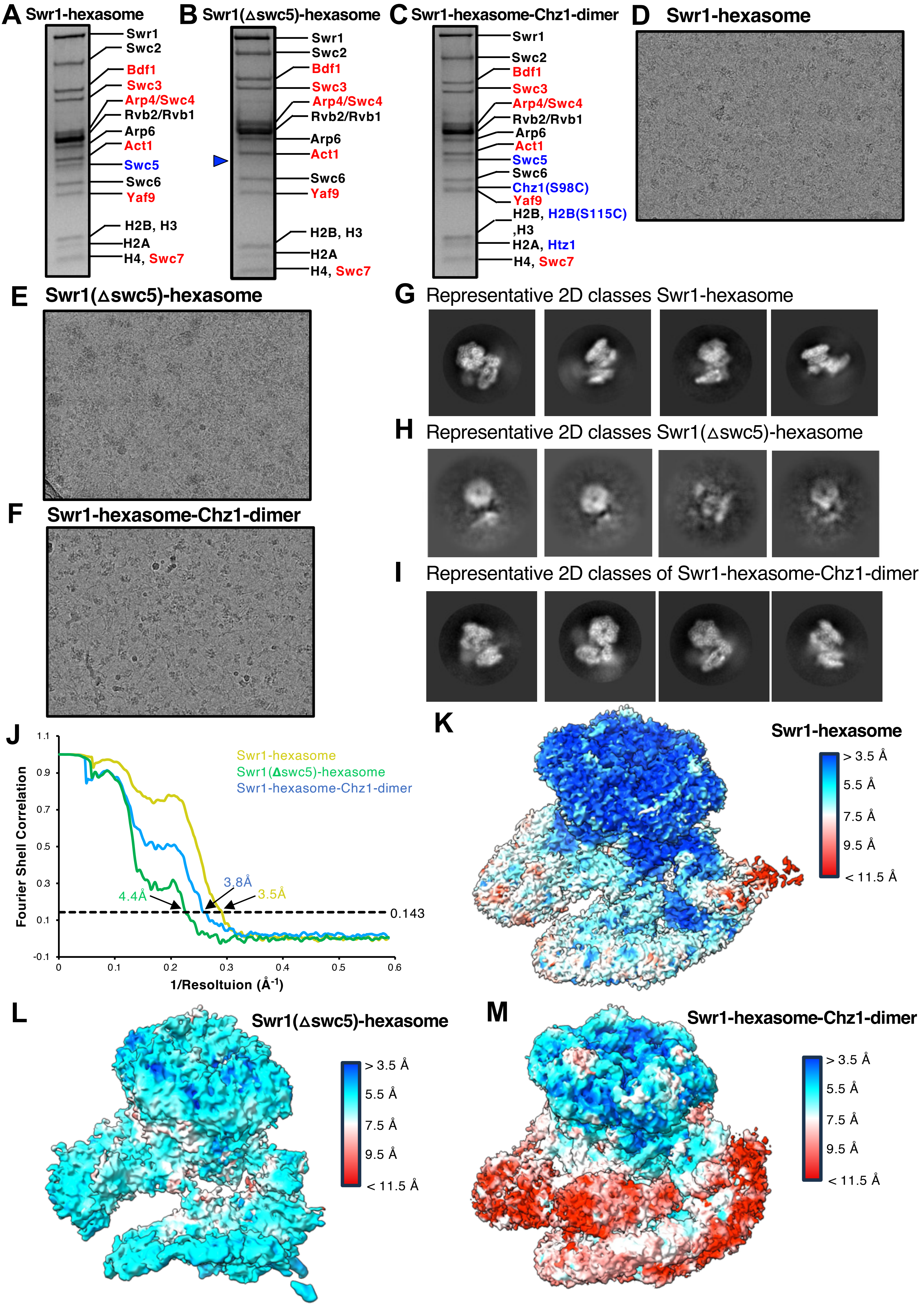
A Swr1-hexasome
Swr1
Swc2
Bdf1
Swc3
Arp4/Swc4
Rvb2/Rvb1
Arp6
Act1
Swc5
Swc6
Yaf9
H2B, H3
H2A
H4, Swc7
B Swr1(Δswc5)-hexasome
Swr1
Swc2
Bdf1
Swc3
Arp4/Swc4
Rvb2/Rvb1
Arp6
Act1
Swc6
Yaf9
H2B, H3
H2A
H4, Swc7
C Swr1-hexasome-Chz1-dimer
Swr1
Swc2
Bdf1
Swc3
Arp4/Swc4
Rvb2/Rvb1
Arp6
Act1
Swc5
Swc6
Chz1(S98C)
Yaf9
H2B, H2B(S115C)
,H3
H2A, Htz1
H4, Swc7
D Swr1-hexasome
E Swr1(Δswc5)-hexasome
F Swr1-hexasome-Chz1-dimer
G Representative 2D classes Swr1-hexasome
H Representative 2D classes Swr1(Δswc5)-hexasome
I Representative 2D classes of Swr1-hexasome-Chz1-dimer
J
Fourier Shell Correlation
1/Resoltuion (Å-1)
Swr1-hexasome
Swr1(Δswc5)-hexasome
Swr1-hexasome-Chz1-dimer
4.4Å
3.8Å
3.5Å
0.143
K Swr1-hexasome
> 3.5 Å
5.5 Å
7.5 Å
9.5 Å
< 11.5 Å
L Swr1(Δswc5)-hexasome
> 3.5 Å
5.5 Å
7.5 Å
9.5 Å
< 11.5 Å
M Swr1-hexasome-Chz1-dimer
> 3.5 Å
5.5 Å
7.5 Å
9.5 Å
< 11.5 Å

Figure S1. Cryo-EM analysis of the 3.5 Å SWR1-hexasome, the 4.4 Å SWR1(△swc5)-hexasome and 3.8 Å SWR-hexasome-Chz1-dimer volumes, related to Figure 1, Figure 3 and Figure 5.

(A) SDS-PAGE analysis of SWR1-hexasome complex that was used for cryo-EM grid preparation. SWR1 subunits that were visible in the cryo-EM structure are labelled in black, while the SWR1 subunits that were present in the sample, but disordered in the structure, are labelled in red. Swc5 (which is ordered in the cryo-EM structure) is labelled in blue to highlight its position on the SDS-PAGE gel. This panel only displays a representative lane from SDS-PAGE analysis prior to samples being selected, pooled and concentrated for grid preparation.

(B) Same as panel A, except the SWR1**(**△swc5) complex was used instead of SWR1. The triangle highlights the position where the missing Swc5 subunit would be expected.

(C) Same as panel A, except Chz1(S98C):Htz1/H2B(S115C) was included when forming the complex. The Chz1(S98C), Htz1/H2B(S115C) labels are coloured in blue to highlight their positions on the SDS-PAGE gel.

(D) Representative micrograph from the SWR1-hexasome dataset.

(E) Representative micrograph from the SWR1(△swc5)-hexasome dataset.

(F) Representative micrograph from the SWR1-hexasome-Chz1-dimer dataset.

(G) Four representative 2D class averages of particles assigned to SWR1-hexasome complex.

(H) Four representative 2D class averages of particles assigned to the SWR1(△swc5) complex.

(I) Four representative 2D class averages of particles assigned to the SWR1-hexasome-Chz1-dimer complex.

(J) gFSC curves of the SWR1-hexasome, SWR1(△swc5)-hexasome, and SWR1-hexasome-Chz1-dimer.

(K) Local resolution of the SWR1-hexasome volume.

(L) Local resolution of the SWR1(△swc5)-hexasome volume.

(M) Local resolution of SWR1-hexasome-Chz1-dimer volume.

FIGURE S2

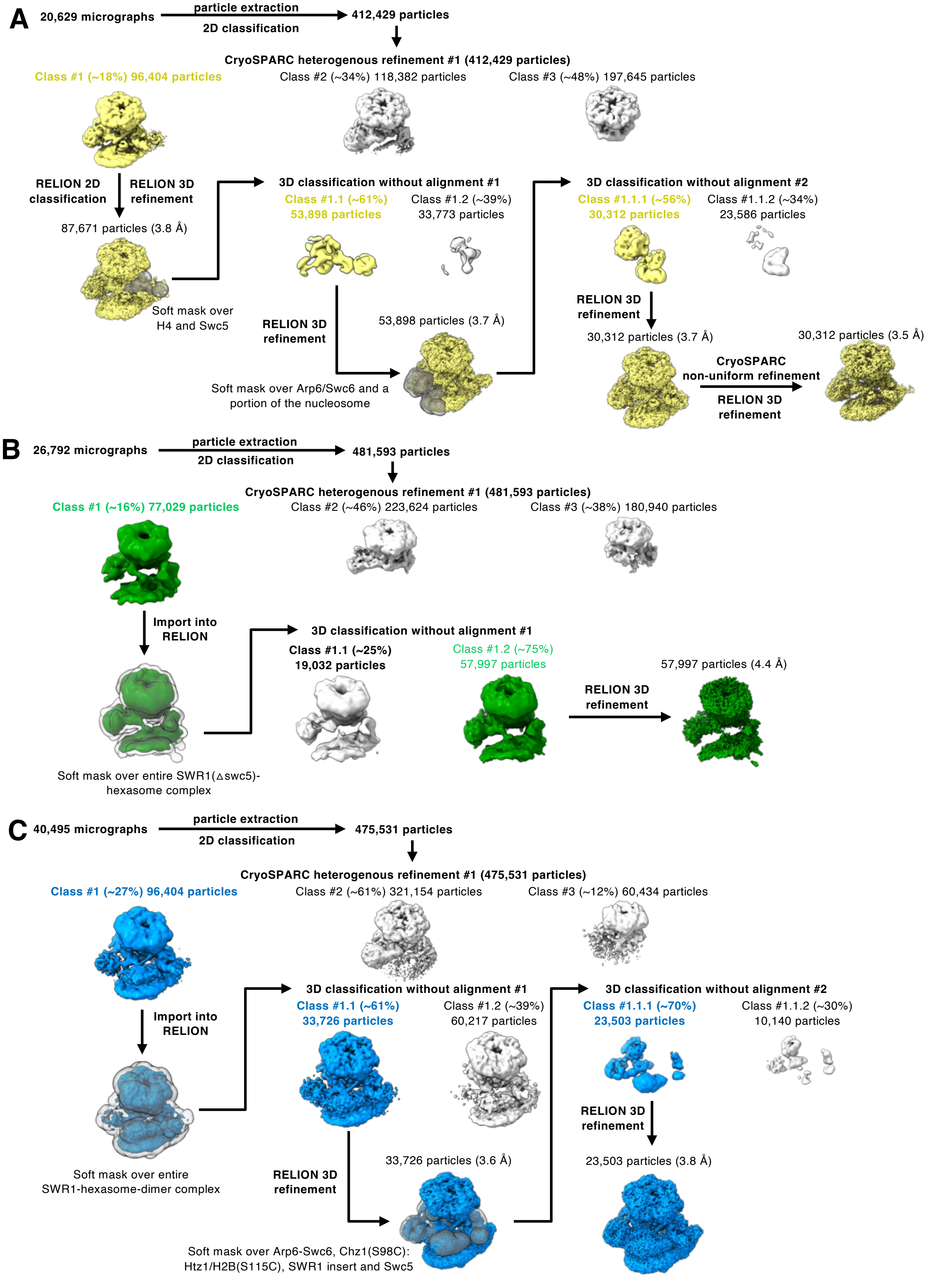
A
20,629 micrographs
particle extraction
2D classification
412,429 particles
CryoSPARC heterogenous refinement #1 (412,429 particles)
Class #1 (~18%) 96,404 particles
Class #2 (~34%) 118,382 particles
Class #3 (~48%) 197,645 particles
RELION 2D classification
RELION 3D refinement
87,671 particles (3.8 Å)
Soft mask over H4 and Swc5
3D classification without alignment #1
Class #1.1 (~61%) 53,898 particles
Class #1.2 (~39%) 33,773 particles
RELION 3D refinement
53,898 particles (3.7 Å)
Soft mask over Arp6/Swc6 and a portion of the nucleosome
3D classification without alignment #2
Class #1.1.1 (~56%) 30,312 particles
Class #1.1.2 (~34%) 23,586 particles
RELION 3D refinement
30,312 particles (3.7 Å)
CryoSPARC non-uniform refinement
RELION 3D refinement
30,312 particles (3.5 Å)
B
26,792 micrographs
particle extraction
2D classification
481,593 particles
CryoSPARC heterogenous refinement #1 (481,593 particles)
Class #1 (~16%) 77,029 particles
Class #2 (~46%) 223,624 particles
Class #3 (~38%) 180,940 particles
Import into RELION
Soft mask over entire SWR1(∆swc5)-hexasome complex
3D classification without alignment #1
Class #1.1 (~25%) 19,032 particles
Class #1.2 (~75%) 57,997 particles
RELION 3D refinement
57,997 particles (4.4 Å)
C
40,495 micrographs
particle extraction
2D classification
475,531 particles
CryoSPARC heterogenous refinement #1 (475,531 particles)
Class #1 (~27%) 96,404 particles
Class #2 (~61%) 321,154 particles
Class #3 (~12%) 60,434 particles
Import into RELION
Soft mask over entire SWR1-hexasome-dimer complex
3D classification without alignment #1
Class #1.1 (~61%) 33,726 particles
Class #1.2 (~39%) 60,217 particles
RELION 3D refinement
33,726 particles (3.6 Å)
Soft mask over Arp6-Swc6, Chz1(S98C): Htz1/H2B(S115C), SWR1 insert and Swc5
3D classification without alignment #2
Class #1.1.1 (~70%) 23,503 particles
Class #1.1.2 (~30%) 10,140 particles
RELION 3D refinement
23,503 particles (3.8 Å)

Figure S2. Cryo-EM analysis of the SWR1-hexasome, SWR1(△swc5)-hexasome and SWR1-hexasome-Chz1-dimer complexes, related to Figure 1 and Figure 5.

(A) Cryo-EM processing workflow of the 3.5Å SWR1-hexasome complex.

(B) Cryo-EM processing workflow of the 4.4Å SWR1(**Δ**swc5)-hexasome complex.

(C) Cryo-EM processing workflow of the 3.8Å SWR1-hexasome-Chz1-dimer complex.

FIGURE S3

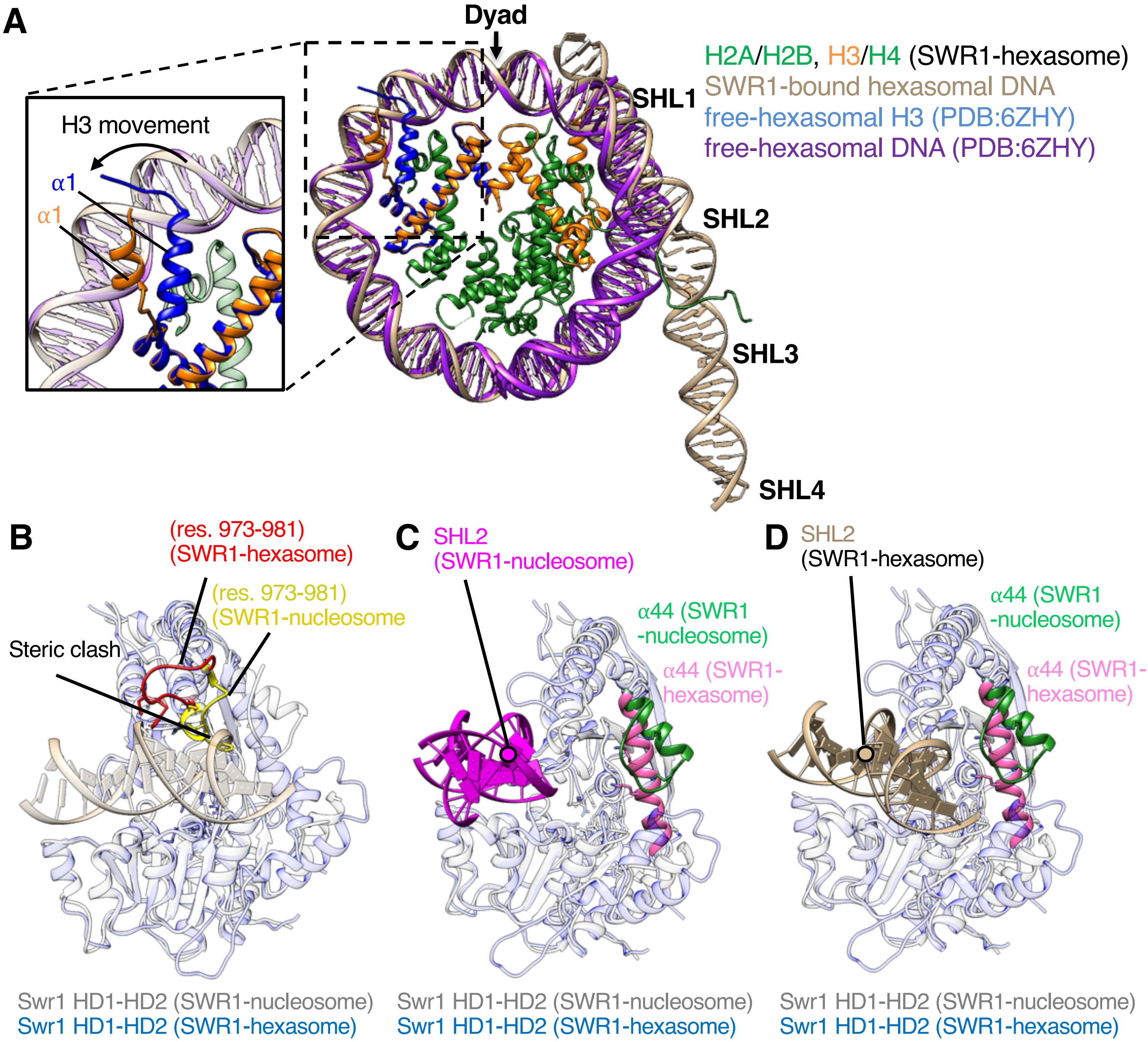

Figure S3. Structural changes in the hexasome and Swr1 motor domains in the SWR1-hexasome complex, related to Figure 1 and Figure 2.

(A) DNA at super helical location (SHL) 2 is unwrapped in the SWR1-bound hexasome structure. The free hexasome (PDB:6ZHY) was superimposed onto the SWR1-bound hexasome using the histone core particle as a reference. For simplicity, in the free hexasome structure, only the built-in coordinates for the DNA and the built-in coordinates of one copy of the built-in coordinates of the H3 histone is shown. The close up view on the left shows α1 helix of histone H3 shifting in position in the SWR1-bound hexasome.

(B) A conformational change in residues (973-981) is required for Swr1 HD1-HD2 to engage the hexasome. A steric clash is seen between this region in the SWR1-nucleosome structure and the unwrapped DNA in the SWR1-bound hexasome structure.

(C) The new DNA binding interface observed in the SWR1-hexasome structure is unable to bind nucleosomal DNA at SHL2 in the SWR1-nucleosome complex.

(D) Conformational change in α44 is required to engage the unwrapped DNA in the SWR1-bound hexasome.

FIGURE S4

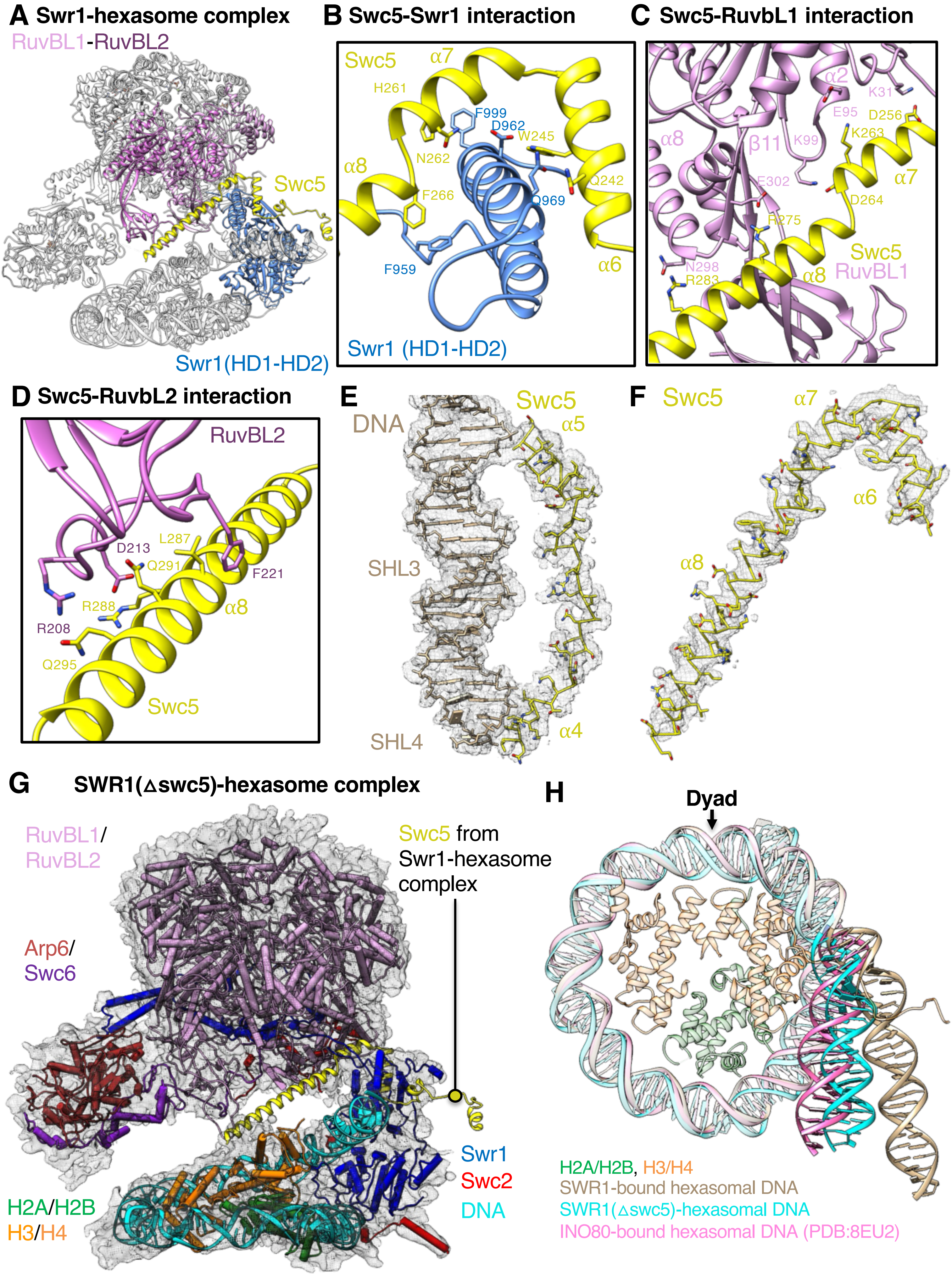

A Swr1-hexasome complex
RuvBL1-RuvBL2
Swc5
Swr1(HD1-HD2)
B Swc5-Swr1 interaction
Swc5
α7
α8
α6
Swr1 (HD1-HD2)
C Swc5-RuvbL1 interaction
α2
α8
β11
α7
Swc5
RuvBL1
D Swc5-RuvbL2 interaction
RuvBL2
α8
Swc5
E
DNA
Swc5
α5
SHL3
SHL4
α4
F
Swc5
α7
α6
α8
G SWR1(∆swc5)-hexasome complex
RuvBL1/
RuvBL2
Arp6/
Swc6
Swc5 from
Swr1-hexasome
complex
Swr1
Swc2
DNA
H2A/H2B
H3/H4
H
Dyad
H2A/H2B, H3/H4
SWR1-bound hexasomal DNA
SWR1(∆swc5)-hexasomal DNA
INO80-bound hexasomal DNA (PDB:8EU2)

Figure S4. Details of interaction between Swc5 and the other subunits of the SWR1 complex, related to Figure 3.

(A) Overview of the Swc5, RuvBL1, RuvBL2, Swr1 (HD1-HD2) interface. The structure of SWR1-hexasome is shown, with the Swc5, RuvBL1, RuvBL2, Swr1 (HD1-HD2) are highlighted.

(B) Detailed view of the Swc5-Swr1 (HD1-HD2) interface.

(C) Detailed view of the Swc5-Ruvb1 interface.

(D) Detailed view of the Swc5-Ruvb2 interface.

(E) Representative density for the assigned DNA-binding region of Swc5 and the unwrapped hexasomal DNA in the SWR1-hexasome complex (contoured at 3σ).

(F) Representative density for α6-α8 of Swc5 in the SWR1-hexasome complex (contoured at 5σ).

(G) Overview of the SWR1(∆swc5)-hexasome complex at 4.4 Å. The SWR1-hexasome structure was superimposed onto the SWR1(∆swc5)-hexasome structure, using the histone core particles as a reference. For clarity, in the SWR1-hexasome structure only Swc5 is shown.

(H) Structural comparison between SWR1(∆swc5)-bound hexasome, SWR1-bound hexasome and INO80-bound hexasome (PDB: 8EU2). Both the SWR1 (∆swc5)-bound hexasome and the INO80-bound hexasome (PDB: 8EU2) were superimposed onto the SWR1-bound hexasome using the histone core particles as a reference. For clarity, in the SWR1(∆swc5)-bound hexasome and the INO80-bound hexasome (PDB: 8EU2), only the hexasomal DNA is shown.

FIGURE S5

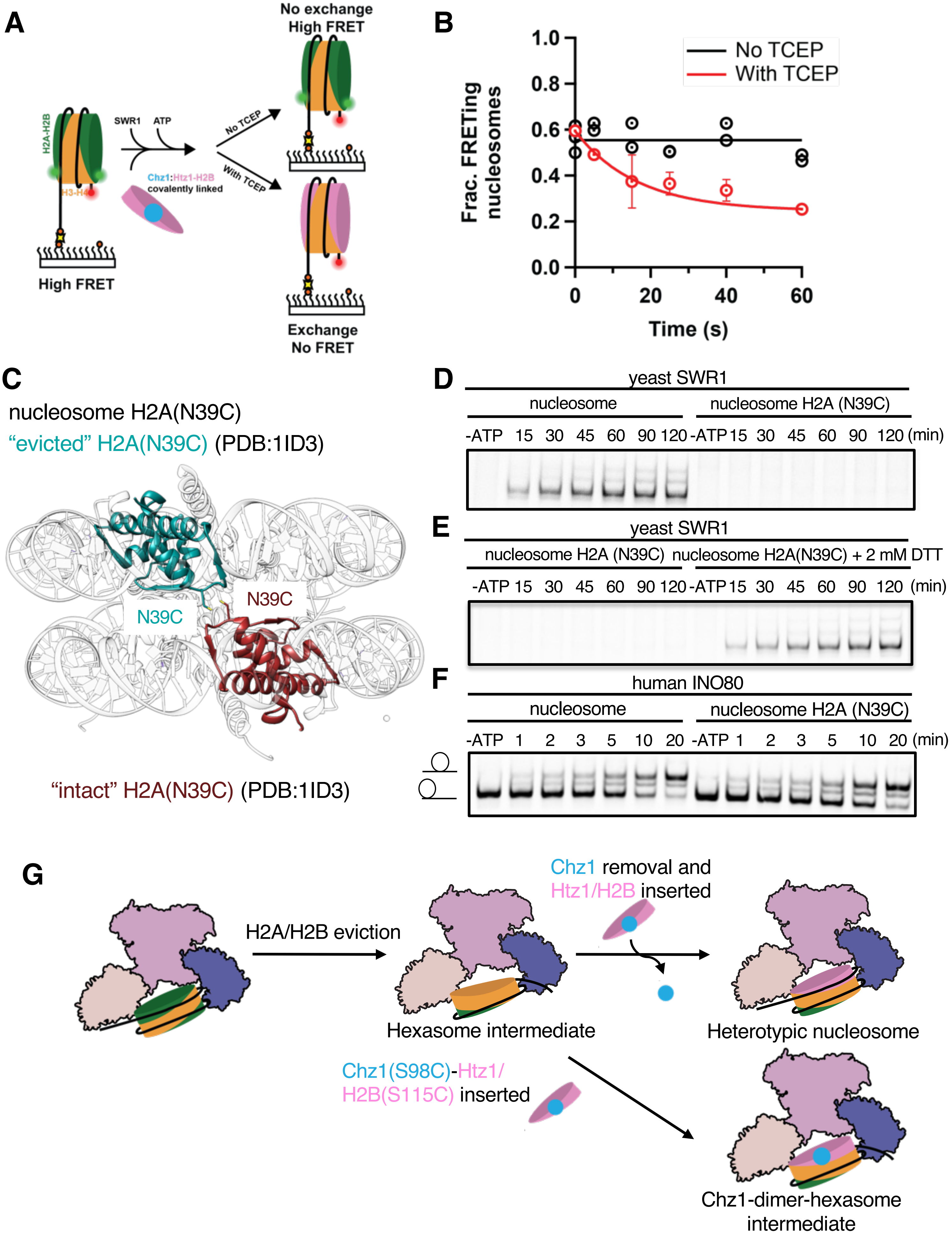

A
No exchange
High FRET
H2A-H2B
SWR1
ATP
No TCEP
With TCEP
Chz1:Htz1-H2B
covalently linked
H3-H4
High FRET
Exchange
No FRET
B
No TCEP
With TCEP
Frac. FRETing nucleosomes
Time (s)
1.0
0.8
0.6
0.4
0.2
0.0
0
20
40
60
C
nucleosome H2A(N39C)
"evicted" H2A(N39C) (PDB:1ID3)
N39C
N39C
"intact" H2A(N39C) (PDB:1ID3)
D
yeast SWR1
nucleosome
nucleosome H2A (N39C)
-ATP 15 30 45 60 90 120 -ATP 15 30 45 60 90 120 (min)
E
yeast SWR1
nucleosome H2A (N39C)
nucleosome H2A(N39C) + 2 mM DTT
-ATP 15 30 45 60 90 120 -ATP 15 30 45 60 90 120 (min)
F
human INO80
nucleosome
nucleosome H2A (N39C)
-ATP 1 2 3 5 10 20 -ATP 1 2 3 5 10 20 (min)
G
H2A/H2B eviction
Chz1 removal and
Htz1/H2B inserted
Hexasome intermediate
Heterotypic nucleosome
Chz1(S98C)-Htz1/
H2B(S115C) inserted
Chz1-dimer-hexasome
intermediate

Figure S5. A covalently linked Chz1(S98C)-Htz1-H2B(115C) inhibits SWR1 exchange, and a covalently linked H2A(N39C)-H2A(N39C) nucleosome inhibits SWR1 exchange, but not INO80 sliding, related to Figure 4 and Figure 5.

(A) Cartoon of smTIRF histone exchange assay. H2A-H2B containing nucleosomes labelled with a FRET donor on H2A and a FRET acceptor on the short end of the DNA linker are immobilized on a microscope slide. SWR1, ATP and a covalently linked Chz1(S98C)-Htz1-H2B(S115C) dimer are flowed in to initiate exchange.

(B) Fraction of FRETing nucleosomes over time. In the presence of TCEP, the covalent bond between Chz1(S98C) and the Htz1-H2B(S115C) dimer is reduced, allowing histone exchange to take place.

(C) The crystal structure of the canonical nucleosome (PDB:1ID3). The point mutations introduced to generate the covalently linked H2A(N39C) is shown. The "evicted" nucleosome represents the proximal H2A whereby density is not visible in the SWR1-hexasome structure, while the "intact" H2A represents the distal H2A whereby density is visible in the SWR1-hexasome structure.

(D) A covalently linked H2A(N39C)-H2A(N39C) nucleosome inhibits SWR1-mediated histone exchange. Gel-based histone exchange assay.

(E) Only in the presence of 2 mM DTT is the covalent bond reduced and SWR1-mediated histone exchange is restored.

(F) A covalently linked H2A(N39C)-H2A(N39C) nucleosome does <u>not</u> inhibit INO80 sliding activity.

(G) Simplified cartoon showing the additional steps (not shown in Figure 1A) involved in the SWR1-mediated histone exchange leading up to the heterotypic nucleosome. Swr1 removes Chz1 from the Chz1-Htz1-H2B complex prior to forming the heterotypic nucleosome. The covalently linked Chz1(S98C)-Htz1-H2B(S115C) prevents SWR1 from forming the heterotypic nucleosome, resulting in a trapped intermediate state

FIGURE S6

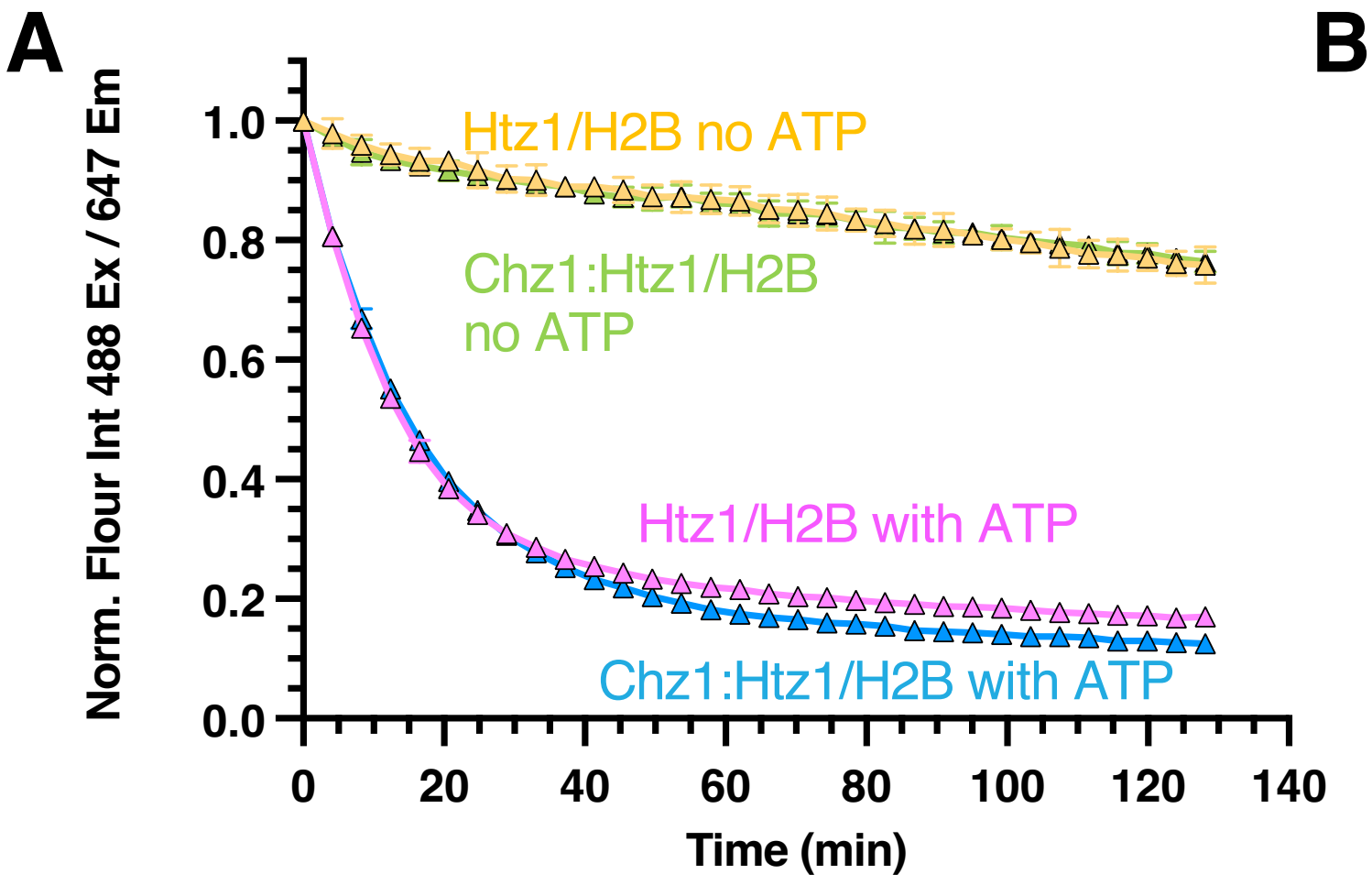

Dimers added	Rate (min^{-1})	Plateau
Htz1/H2B	0.0644 ± 0.0016	0.1862 ± 0.0063
Chz1:Htz1/H2B	0.0551 ± 0.0002	0.1389 ± 0.0058

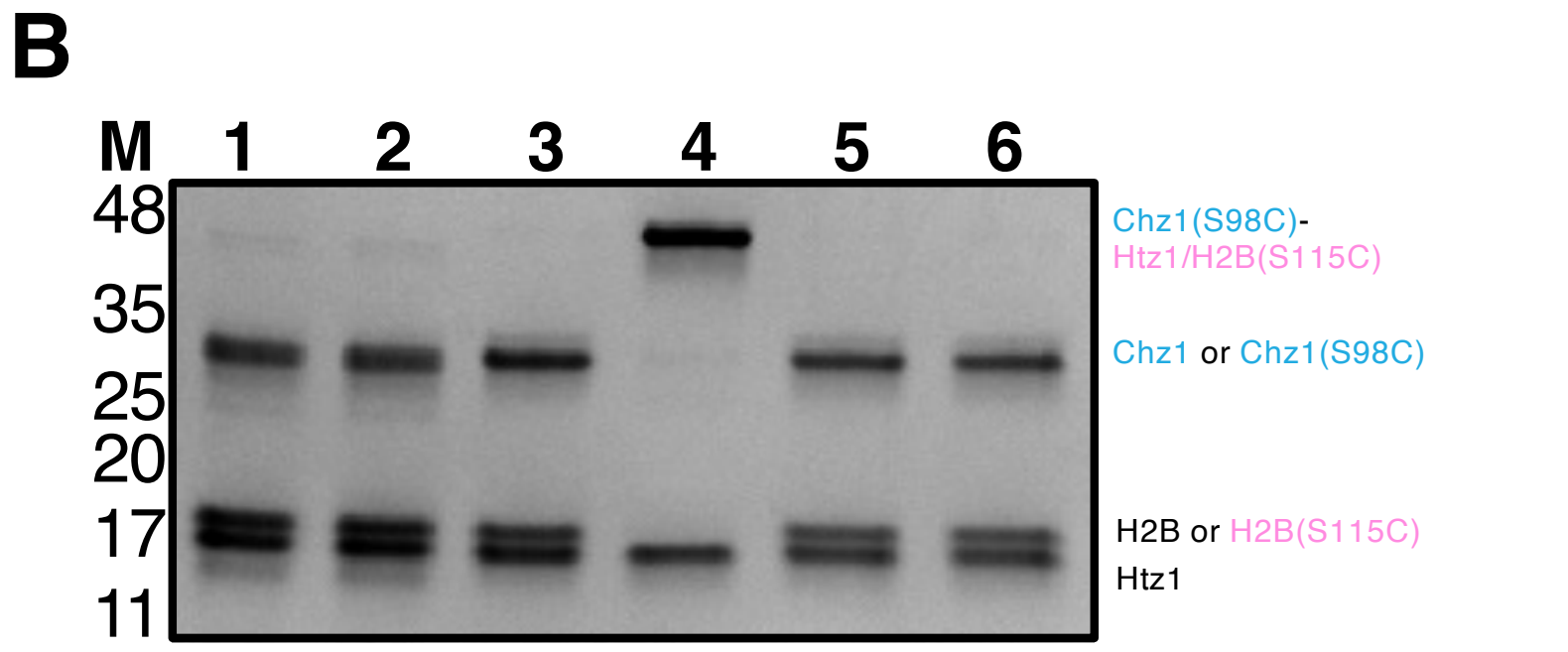

Lanes:

1. Chz1-Htz1-H2B complex in a reducing GLB
2. Chz1-Htz1-H2B complex in a non-reducing GLB
3. Chz1(S98C)-Htz1-H2B(S115C) in a reducing GLB
4. Chz1(S98C)-Htz1-H2B(S115C) in a non-reducing GLB
5. Pre-incubated Chz1(S98C)-Htz1-H2B(S115C) in 2mM DTT in a reducing GLB
6. Pre-incubated Chz1(S98C)-Htz1-H2B(S115C) in 2mM DTT in a non-reducing GLB

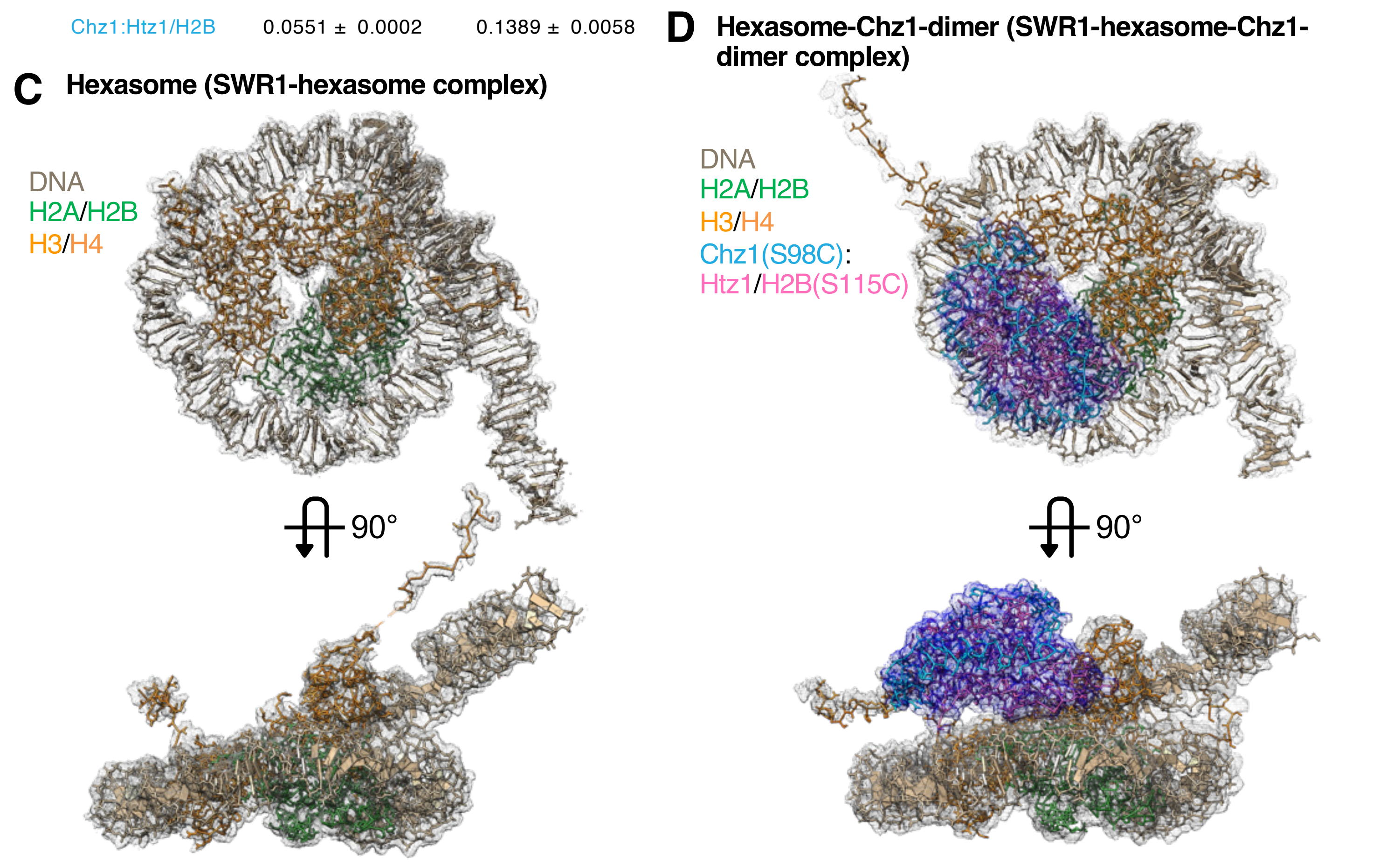

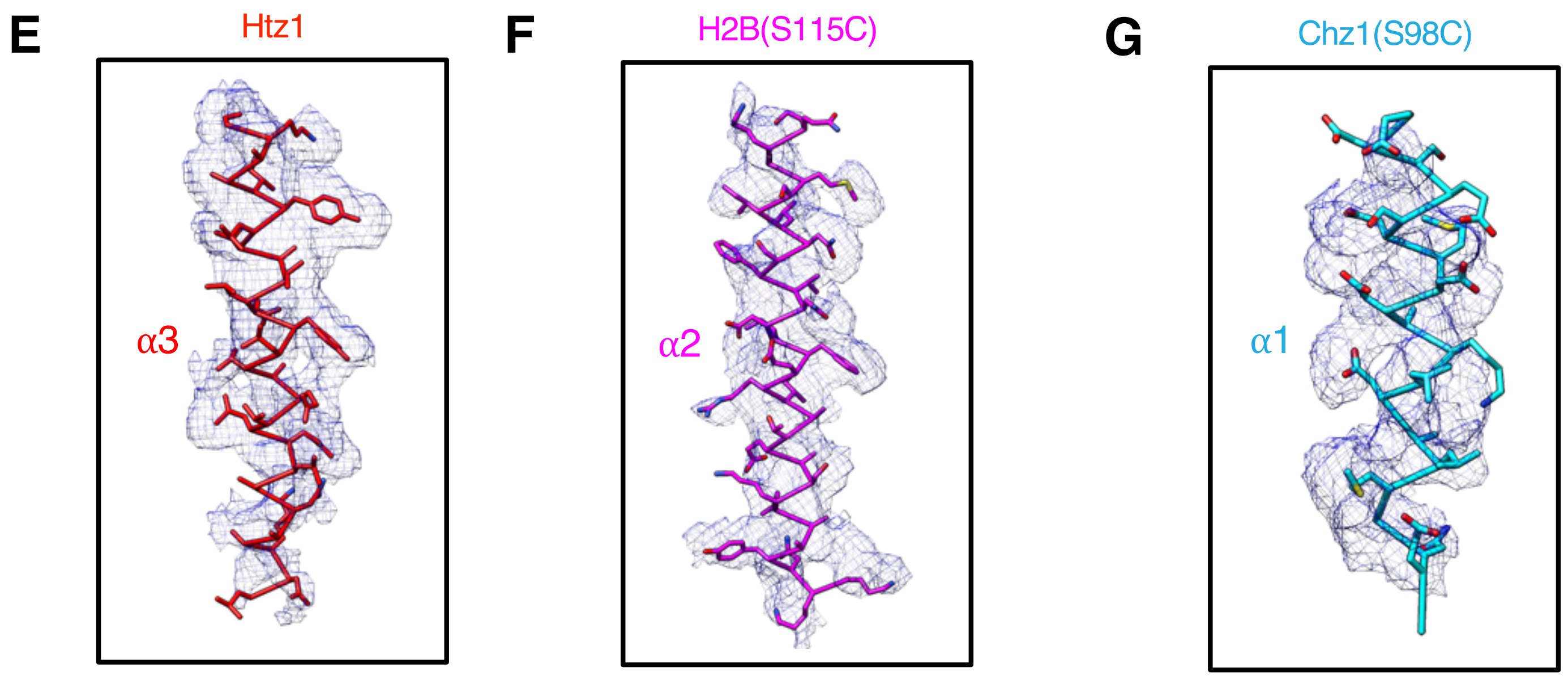

Figure S6. Chaperone bound Htz1/H2B (Chz1:Htz1/H2B) does not influence the rate of SWR1-mediated histone exchange, and representative density volumes confirm the assignment of the Chz1(S98C):Htz1/H2B(S115C) in the SWR1-hexasome-Chz1-dimer structure, related to Figure 4 and Figure 5.

(A) SWR1 exchange with Htz1/H2B dimers or pre-complexed Chz1-Htz1-H2B showed that the presence of Chz1 does not influence the rate or completion of the exchange reaction catalysed by SWR1.

(B) Chz1(S98C) forms a covalent bond with Htz1/H2B(S115C). The covalent bond between Chz1(S98C) and Htz1/H2B(S115C) is reduced in the presence of DTT.

(C) Representative density for the hexasome in the SWR1-hexasome complex (contoured at 3σ).

(D) Representative density for the hexasome in the SWR1-hexasome-Chz1-dimer complex (contoured at 3σ). The density for Chz1(S98C):Htz1/H2B(S115C) is shown in blue.

(E) Representative density for α3 of Htz1 in the SWR1-hexasome-Chz1-dimer structure (contoured at 6σ).

(F) Representative density for α2 of H2B(S115C) in the SWR1-hexasome-Chz1-dimer structure (contoured at 6σ).

(G) Representative density for α1 of Chz1(S98C) in the SWR1-hexasome-Chz1-dimer structure (contoured at 5σ).

FIGURE S7

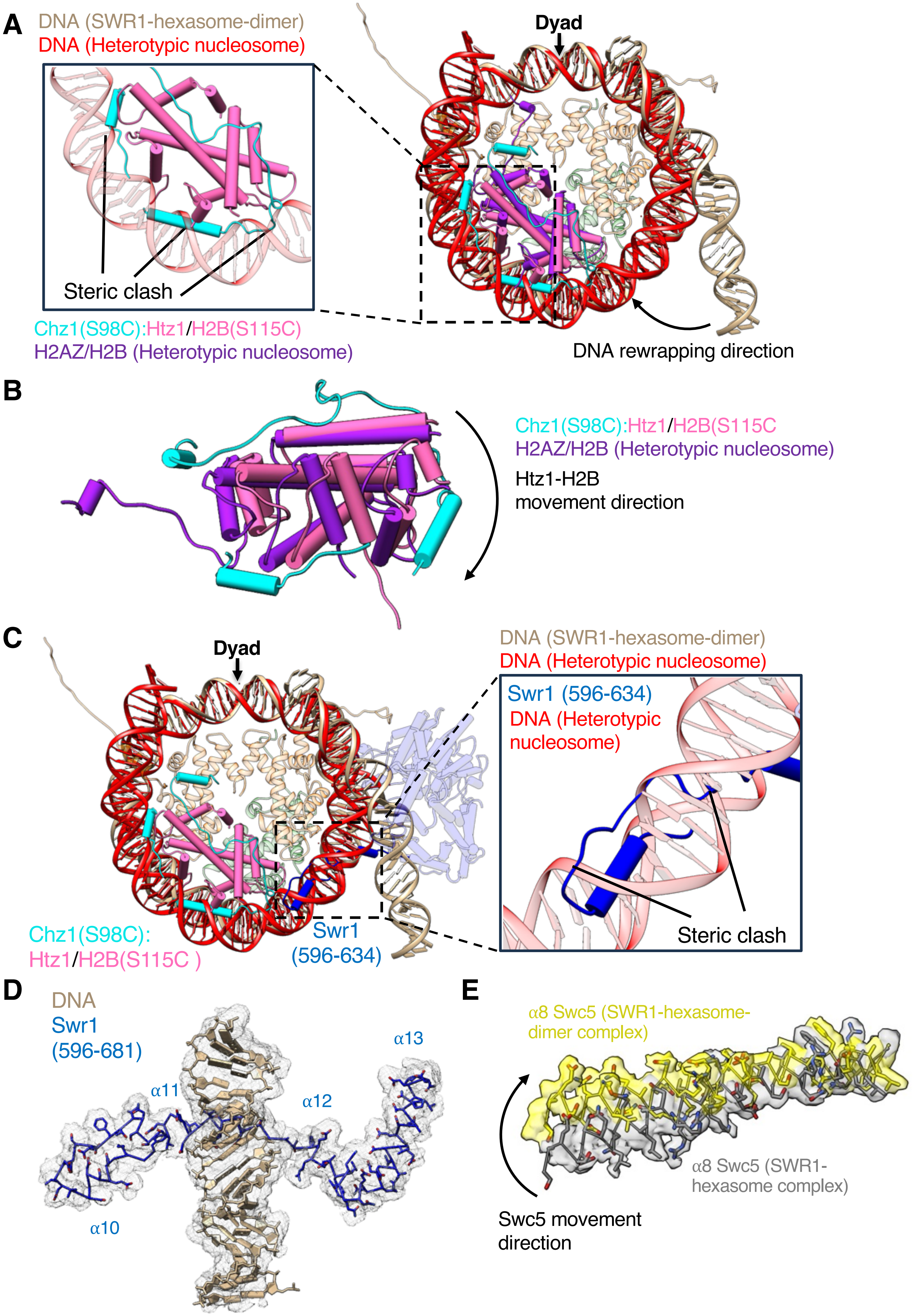
A
DNA (SWR1-hexasome-dimer)
DNA (Heterotypic nucleosome)
Steric clash
Chz1(S98C):Htz1/H2B(S115C)
H2AZ/H2B (Heterotypic nucleosome)
Dyad
DNA rewrapping direction
B
Chz1(S98C):Htz1/H2B(S115C
H2AZ/H2B (Heterotypic nucleosome)
Htz1-H2B
movement direction
C
Dyad
DNA (SWR1-hexasome-dimer)
DNA (Heterotypic nucleosome)
Swr1 (596-634)
DNA (Heterotypic nucleosome)
Steric clash
Chz1(S98C):
Htz1/H2B(S115C)
Swr1
(596-634)
D
DNA
Swr1
(596-681)
α11
α12
α13
α10
E
α8 Swc5 (SWR1-hexasome-dimer complex)
α8 Swc5 (SWR1-hexasome complex)
Swc5 movement direction

Figure S7. DNA rewrapping to form a heterotypic nucleosome is impaired in the SWR1-hexasome-Chz1-dimer structure, related to Figure 5 and Figure 6.

(A) Structural comparison between the SWR1-bound hexasome-dimer and an free heterotypic nucleosome. The heterotypic nucleosome (PDB:5B31) is superimposed onto the SWR1-bound hexasome-dimer complex. For simplicity, only the built-in coordinates of the re-wrapped DNA in the heterotypic nucleosome are shown. The close up view shown to the left highlights the steric clash that occurs between the Chz1(S98C) and the re-wrapped DNA in the heterotypic nucleosome.

(B) Structural comparison between SWR1-bound hexasome-Chz1-dimer and an apo-heterotypic nucleosome. The Chz1(S98C)-Htz1-H2B(S115C) has not fully been deposited into the hexasome to form the heterotypic nucleosome. The direction of Htz1-H2B movement is shown.

(**C)** The Swr1 subunit prevents DNA rewrapping required to form the heterotypic nucleosome. The inset if a close of view highlighting the steric clash between Swr1 (596-634) and the rewrapped DNA in the heterotypic nucleosome.

(D) Representative density for the residues 596-681 of Swr1 and the unwrapped hexasomal DNA in the SWR1-hexasome-Chz1-dimer complex (contoured at 3σ).

(E) Representative density for α8 of Swc5 in the SWR1-hexasome and SWR1-hexasome-Chz1-dimer complex (contoured at 3σ). The movement direction of Swc5 is shown.

TABLE S1 – Cryo-EM data collection, refinement and statistics, related to Figure 1, Figure 3 and Figure 5

	SWR1-hexasome complex	SWR1(Δswc5)-hexasome complex	SWR1-hexasome-Chz1-dimer complex
**Data collection and processing**			
Magnification	105,000	105,000	105,000
Voltage (kV)	300	300	300
Defocus range	-0.7 to -1.9	-0.7 to -1.9	-0.7 to -1.9
Pixel size	0.85	0.85	0.85
Symmetry imposed	C1	C1	C1
Initial particle images	412,429	481,593	475,531
Final particle images	30,312	57,997	23,503
Map resolution (Å)	3.5	4.4	3.8
FSC threshold	0.143	0.143	0.143
**Refinement**			
Initial model used (PDB code)	6GEJ	6GEJ	6GEJ, 2JSS, 6AE8
Model resolution (Å)	3.5	4.4	3.8
Map Sharpening *B* factor ($Å^2$)	-20	-20	15
**Fit to map**			
Refinement Program	Phenix	Phenix	Phenix
Map CC (mask)	0.82	0.50	0.78
Map CC (peaks)	0.70	0.30	0.65
Map CC (volume)	0.80	0.52	0.77
Map CC, Swr1	0.77	0.65	0.69
Map CC $(RuvBL1/RuvBL2)_3$	0.87	0.67	0.87
Map CC, Arp6	0.50	0.69	0.48
Map CC, Swc6	0.50	0.50	0.52
Map CC, Swc2	0.51	0.66	0.62
Map CC, Swc5	0.71	-	0.70
Map CC, Hexasome	0.63	0.50	0.74
Map CC, Chz1(S98C):Htz1/H2B(S115C)	-	-	0.52
**Model composition**			
Database ID codes	PDB: 8QYV EMD: 18764	PDB : 9FBW EMD: 50297	PDB: 8QZ0 EMD: 18769
Non-hydrogen atoms	41998	39683	44167
Protein residues	4795	4522	5092
Nucliec acid residues	236	224	226
Ligands	20	20	20
**R.m.s. devitaions**			
Bond lengths (Å)	0.003	0.003	0.003
Bond angles (°)	0.641	0.675	0.682
**Validation**			
Clashscore	12.27	19.24	16.41
Rotamer outliers (%)	0.43	0.00	0.38
**Ramachandran plot**			
Favored (%)	93.69	92.38	93.51
Allowed (%)	6.02	7.40	6.15
Disallowed (%)	0.30	0.22	0.34
